# Comparative analysis of serum antibody responses and immune landscapes in patients with acute DENV-1, DENV-2, and Chikungunya virus infections

**DOI:** 10.3389/fimmu.2026.1948489

**Published:** 2026-10-28

**Authors:** Ying Zhang, Shengze Zhang, Longyu Zou, Haolu Zha, Weijian Tian, Pengjie Ma, Yachao Zuo, Caijun Sun, Yuelong Shu, Jianhui Yuan, Yanping Ma, Huanle Luo, Nan Wu

**Affiliations:** 1 Shenzhen Nanshan Center for Disease Control and Prevention, Shenzhen, China; 2 School of Public Health (Shenzhen), Shenzhen Key Laboratory of Pathogenic Microbes and Biosafety, Shenzhen Campus of Sun Yat-sen University, Shenzhen, China; 3 Key Laboratory of Tropical Disease Control (Sun Yat-sen University), Ministry of Education, Guangzhou, China; 4 Beijing Research Center for Respiratory Infectious Diseases, Beijing, China; 5 Key Laboratory of Pathogen Infection Prevention and Control (MOE), State Key Laboratory of Respiratory Health and Multimorbidity, National Institute of Pathogen Biology, Chinese Academy of Medical Sciences & Peking Union Medical College, Beijing, China

**Keywords:** antibody responses, chikungunya virus, cytokine profiling, dengue virus, host immune responses, viral RNA levels

## Abstract

**Introduction:**

Dengue virus (DENV) and chikungunya virus (CHIKV) are major mosquito-borne arboviruses that increasingly co-circulate in tropical and subtropical regions, including southern China. However, the extent to which these viruses induce shared or distinct host immune responses remains poorly understood. This study aimed to comparatively characterize immune responses during acute DENV-1, DENV-2, and CHIKV infections.

**Materials and methods:**

A total of 80 serum samples were analyzed, including 65 samples from patients with laboratory-confirmed DENV-1 (n = 29), DENV-2 (n = 10), or CHIKV (n = 26) infections and 15 healthy controls.Viral RNA was detected by real-time reverse transcription polymerase chain reaction. Virus-specific IgM and IgG responses were measured by Enzyme-Linked Immunosorbent Assay, and serum cytokines were quantified using a multiplex assay. Group comparisons used nonparametric tests, and associations among antibody responses, cytokine concentrations, and viral RNA levels were assessed using Spearman’s rank correlation. Multiple comparisons were adjusted using the Benjamini-Hochberg (BH) procedure.

**Results:**

The three infections shared features of interferon-associated immune activation and relatively restrained systemic inflammation but exhibited distinct immune mediator profiles, characterized by differential inflammatory mediator and growth factor responses. After multiple-testing correction, cytokine–cytokine correlation analysis revealed both shared and infection-specific patterns of immune mediator covariation, with shared associations involving IL-9, TNF-β, CCL4, and CXCL1 across all three infection groups. Positive associations between pathogen-specific IgM responses and Ct values were observed across the infections, although the association was not statistically significant for DENV-2 after multiple-testing correction. IgG responses showed a similar association only in DENV-1 infection. Integration of cytokine and antibody profiles further revealed distinct immune association patterns: DENV-1 showed broader associations involving viral RNA levels, inflammatory mediators, and humoral responses, whereas CHIKV displayed a more restricted pattern centered on associations between inflammatory mediators and IgM responses.

**Discussion:**

These findings reveal shared and distinct immune responses during acute DENV-1, DENV-2, and CHIKV infections and provide a comparative framework for investigating virus-associated immune regulation during acute arboviral infections.

## 1 Introduction

Dengue virus (DENV) is a positive-sense single-stranded RNA virus belonging to the genus *Flavivirus* within the family *Flaviviridae* and comprises four antigenically distinct serotypes (DENV-1-4) (1). Chikungunya virus (CHIKV) is also a positive-sense single-stranded RNA virus and belongs to the genus *Alphavirus* within the family *Togaviridae* (2). Both viruses are primarily transmitted to humans through the bites of infected *Aedes* mosquitoes, causing dengue fever (DF) and chikungunya fever (CHIKF), respectively. As two of the most important mosquito-borne viral diseases worldwide, DENV and CHIKV are widely distributed in tropical and subtropical regions, with their global disease burden increasing steadily in recent years (3–5). In China, DENV incidence has shown a sustained upward trend, particularly in the southern provinces (6), while Guangdong Province experienced the largest recorded local outbreak of CHIKF in 2025 (7). The increasing co-circulation of DENV and CHIKV, particularly in southern China, presents substantial public health challenges because these infections share similar clinical manifestations, complicating differential diagnosis and clinical management. Both DENV and CHIKV infections exhibit a broad clinical spectrum, ranging from asymptomatic or self-limiting febrile illness to more severe disease (8). Severe DENV infection may be characterized by plasma leakage and hemorrhagic manifestations (9). In contrast, CHIKV infection is primarily associated with acute febrile illness and debilitating, sometimes persistent, arthralgia (10). Increasing evidence indicates that disease severity is determined not only by viral replication but also by the host immune response (11). During acute infection, cytokines and chemokines orchestrate the inflammatory response and contribute to both antiviral immunity and immunopathology. In DENV, dysregulated production of pro-inflammatory and regulatory cytokines is closely associated with disease progression, and dynamic changes in mediators such as IL-6, IL-8, IL-10, and TNF-α have been associated with clinical outcomes (12, 13). Likewise, previous studies have shown that CHIKV infection induces robust inflammatory responses characterized by increased levels of TNF-α, CCL2, IFN-γ and IL-6 (14–16). Moreover, elevated CXCL9, CXCL10, and virus-specific IgG have been associated with disease severity and chronic arthralgia (17). Despite these observations, most studies have focused on individual viral infections, and whether DENV and CHIKV induce shared or distinct inflammatory responses remains incompletely understood.

Besides, the humoral immune response also plays a pivotal role in controlling acute arboviral infections. Virus-specific IgM antibodies appear early after infection and serve as important diagnostic markers, whereas IgG antibodies develop subsequently and contribute to long-term immune protection (18, 19). Previous studies have reported associations between viral load and the magnitude or kinetics of pathogen-specific antibody responses. In DENV, combined detection of NS1 antigen, viral load, IgM, and IgG improves disease diagnosis and may facilitate the prediction of severe disease (20). In CHIKV infection, virus-specific IgM antibodies can persist for prolonged periods after acute infection, suggesting sustained humoral immune activation (21). Animal studies have demonstrated that B cells and CHIKV-specific IgM and IgG antibodies are essential for controlling CHIKV infection and facilitating viral clearance (22). In addition, increasing evidence suggests extensive crosstalk between humoral immunity and the inflammatory microenvironment during viral infection. In DENV, cytokines such as IL-2, IL-4, and IL-10 regulate B-cell activation and antibody production, while inflammatory mediators have been reported to correlate with virus-specific IgM responses, with IgM levels negatively correlating with IFN-α, IL-1RA, and MCP-1, and positively correlating with TNF-α, VEGF-D, and SDC-1 (23–25). Moreover, anti-NS1 antibodies can stimulate endothelial cells to produce inflammatory mediators, including IL-6, IL-8, and MCP-1 (26). Similarly, in CHIKV infection, IL-4 has been reported to correlate positively with anti-CHIKV IgM responses, whereas IL-1β shows a negative association with anti-CHIKV IgG levels (27).

Although substantial progress has been made in understanding the immunopathogenesis of DENV and CHIKV infections individually, comparative studies of host immune responses to these clinically important arboviruses remain limited. Previous comparative studies have characterized shared and virus-specific cytokine responses in acute DENV and CHIKV infections (28, 29). However, an integrated characterization of inflammatory responses, pathogen-specific humoral immunity, and viral RNA levels across DENV-1, DENV-2, and CHIKV remains limited. Integrating these immune features may provide a more comprehensive characterization of shared and virus-associated patterns of host immune responses during acute arboviral infection.

In the present study, we performed a comprehensive immunological analysis of serum samples collected from patients with acute DENV-1, DENV-2, and CHIKV infections during the 2025 outbreak in Shenzhen, China. We systematically compared pathogen-specific IgG and IgM responses, serum cytokine and chemokine profiles, and their associations with viral RNA levels inferred from cycle threshold (Ct) values. In addition, we evaluated the influence of sex on humoral immune responses and inflammatory landscapes. Our findings provide a comparative overview of immune responses elicited by three clinically important arboviral infections and identify both shared and virus-associated immunological features.

## 2 Materials and methods

### 2.1 Study population and ethical statement

A total of 80 serum samples were collected, including 65 samples from patients with laboratory-confirmed infections of DENV-1, DENV-2, and CHIKV, and 15 samples from healthy controls. All samples were obtained from the Shenzhen Nanshan Center for Disease Control and Prevention and stored at -80 °C until further analysis. The infected cohort consisted of 29 patients with DENV-1 infection, 10 patients with DENV-2 infection, and 26 patients with CHIKV infection. All infected cases were identified during the acute phase through routine surveillance, and no severe cases were included according to available surveillance records.

All experiments were conducted in accordance with relevant ethical guidelines and regulations and were approved by the Ethics Committee of the Nanshan Center for Disease Control and Prevention (Nanshan Health Supervision Institute), Shenzhen, China (Approval No. 2026A018).

### 2.2 Proteins

The non-structural protein NS1 of DENV-1 (SinoBiological, 40527-V07H), the non-structural protein NS1 of DENV-2 (40263-V07H), and the envelope protein E2 of CHIKV (SinoBiological, 40440-V08B) were all purchased from SinoBiological.

### 2.3 Real-time reverse transcription polymerase chain reaction

Viral RNA was extracted from serum samples using the Nucleic Acid Extraction Kit (Tianlong Biotechnology Co., Ltd., China; Cat. No. T183). The extracted RNA was subsequently subjected to quantitative reverse transcription polymerase chain reaction (qRT-PCR) using the Dengue Virus/Chikungunya Virus Nucleic Acid Detection Kit (Tianlong Biotechnology Co., Ltd., China; Cat. No. P2177). Samples with a cycle threshold (Ct) value < 37 were considered positive. Samples with no Ct value or a Ct value ≥ 37 were considered negative. Each assay was considered valid only when the negative control, positive control, and internal control satisfied the predefined quality control criteria. For DENV-positive samples, viral serotyping was subsequently performed using a commercial multiplex real-time RT-PCR assay targeting DENV-1-4 (BioGerm, Qingdao, China; Cat. No. SL-BM-401-1/2), according to the manufacturer’s instructions. Samples showing a characteristic amplification curve and a Ct value ≤ 35 were considered positive for the corresponding DENV serotype, whereas samples with Ct values > 35 or without detectable amplification were considered negative.

### 2.4 Enzyme-linked immunosorbent assay

Serum levels of IgG and IgM antibodies against DENV-1 NS1, DENV-2 NS1, and CHIKV E2 proteins were detected using an indirect enzyme-linked immunosorbent assay (ELISA). Briefly, 96-well ELISA plates were coated separately with DENV-1 NS1, DENV-2 NS1, or CHIKV E2 proteins at 5 ng/well and incubated overnight at 4 °C. The plates were then blocked with 200 μL/well of 2% bovine serum albumin (BSA; Sigma, V900933) in phosphate-buffered saline (PBS; Biosharp, BL601A) containing 0.05% Tween-20 (Beyotime, ST1726) for 1 hour (h) at room temperature (RT). Serum samples were serially diluted threefold in blocking buffer, and 100 μL of diluted samples ranging from 1:100 to 1:24300 were added to the plates, followed by incubation for 2 h at RT. After washing six times with 0.05% PBST, horseradish peroxidase (HRP)-conjugated goat anti-human IgG (Abcam, ab6858) and IgM antibodies (Abcam, ab97205) diluted 1:20000 were added and incubated for 1 h at RT. The plates were then washed six times with 0.05% PBST, followed by incubation with TMB substrate (MAIGE, RC20160) for 25 min at RT. The reaction was terminated by adding stop solution (Solarbio, C1058), and optical density (OD) was measured at 450 nm using a Multiskan SkyHigh Microplate Reader (ThermoFisher, USA).

### 2.5 Cytokine Bio-Plex

Cytokine concentrations were measured using a 48-plex Bio-Plex Pro Human Cytokine Assay (Bio-Rad, 12007283) according to the manufacturer’s instructions. Briefly, serum samples were diluted 1:3 in sample dilution buffer and incubated with magnetic beads for 1 h. The beads were then washed three times with washing buffer, followed by incubation with detection antibodies for 30 min. After washing, streptavidin-phycoerythrin was added and incubated with the beads for 10 min. Cytokine levels were detected using a Bio-Plex 200 system (Bio-Rad, USA). Of the 48 cytokines initially measured, IFN-α2, IL-5, and VEGF had detection rates of < 10% in each of the three viral infection groups and were excluded from subsequent analyses. The remaining 45 cytokines were detected in all three infection groups and were therefore included in the statistical analysis.

### 2.6 Statistics

All continuous variables were expressed as median (interquartile range, IQR), and categorical variables were presented as counts and percentages. The Kruskal-Wallis test was used for comparisons among multiple groups, followed by Dunn’s multiple comparisons test with Bonferroni correction for multiple comparisons. The Mann-Whitney U test was applied for comparisons between two groups. Correlations between antibody binding levels detected by ELISA and cytokine concentrations were assessed using Spearman’s rank correlation coefficient. *P* values were adjusted for multiple comparisons using the Benjamini-Hochberg (BH) method to control the false discovery rate (FDR). For cytokine-antibody correlation analyses, each infection group was treated as a separate hypothesis family, with 45 cytokines and two antibody measurements resulting in 90 tests per family. For cytokine-cytokine correlation analyses, each infection group was treated as a separate hypothesis family, with 990 pairwise correlation tests per family based on 45 cytokines. For other cytokine-related analyses, including cytokine-Ct correlations, comparisons of cytokine concentrations between sexes, and comparisons between low- and high-Ct groups, each infection group was treated as a separate hypothesis family, with 45 cytokines corresponding to 45 tests per family. For antibody-related analyses, including antibody-Ct correlations, comparisons between low- and high-Ct groups, and comparisons between sexes, the two antibody measurements across the three infection groups were corrected together, resulting in six tests per hypothesis family. A two-tailed *P* value < 0.05 was considered statistically significant. Statistical significance was further denoted as follows: *P < 0.05, **P < 0.01, ***P < 0.001, and ****P < 0.0001. Statistical analyses of clinical data were performed using SPSS Statistics version 25 (IBM, Armonk, NY, USA), while experimental data were analyzed using GraphPad Prism version 8. Python version 3.13 was used for BH multiple-testing correction and for verification of the adjusted *P* values.

## 3 Results

### 3.1 Clinical and virological characteristics of DENV-1, DENV-2, and CHIKV infections

A total of 80 serum samples were collected by the Nanshan District Center for Disease Control and Prevention (Shenzhen, China) between August and November 2025, including 29 DENV-1-positive, 10 DENV-2-positive, and 26 CHIKV-positive cases, together with 15 healthy controls. Infection status was confirmed by viral nucleic acid testing, while all healthy controls tested negative. The male-to-female ratios were 1.23 (55.2% and 44.8%), 1.00 (50.0% and 50.0%), 1.36 (57.7% and 42.3%), and 6.52 (86.7% and 13.3%) in the DENV-1, DENV-2, CHIKV and Control groups, respectively (**Figure 1A**). The median ages (interquartile range, IQR) were 37.00 (30.00-47.00), 51.50 (40.75-65.00), and 36.50 (25.50-47.50) years for patients with DENV-1, DENV-2, and CHIKV infection, respectively, compared with 53.00 (30.50–59.00) years in the Control group (**Figure 1B**). The median Ct values (IQR) for the DENV-1, DENV-2, and CHIKV groups were 17.00 (16.00-23.00), 17.61 (13.13-18.57), and 20.14 (18.00-25.08), respectively (**Figure 1C**). Within each infection group, Ct values did not differ between male and female patients (**Figure 1D**), nor were they correlated with age (**Figures 1E–G**), suggesting that Ct value was not substantially influenced by demographic characteristics in this cohort.

**Figure 1:**
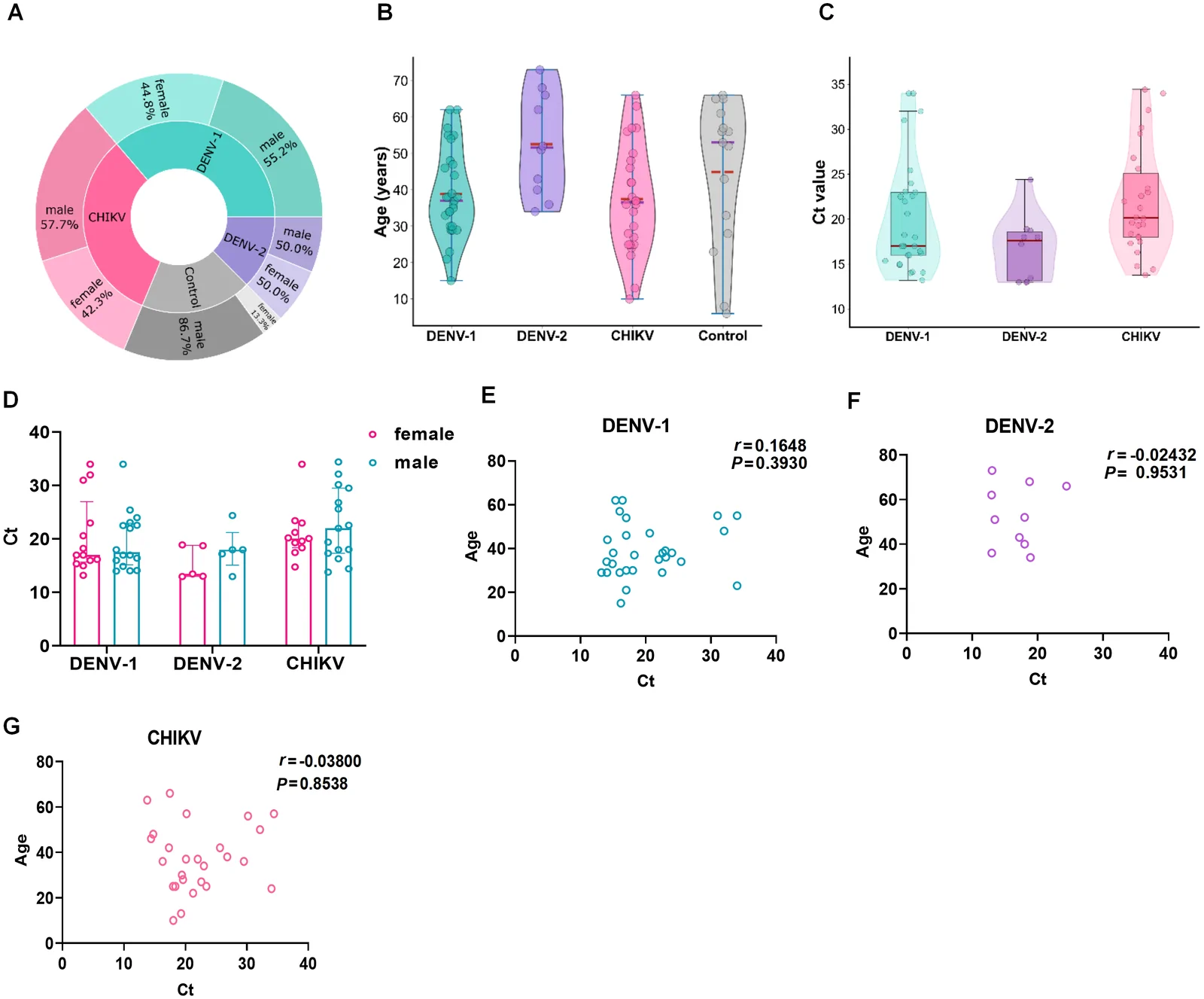
Demographic characteristics and Ct values profiles of DENV-1-, DENV-2-, and CHIKV-infected patients. **(A)** Sex distribution among healthy controls and patients infected with DENV-1, DENV-2, or CHIKV. **(B)** Age distribution among healthy controls and patients infected with DENV-1, DENV-2, or CHIKV. **(C)** Distribution of RT-PCR cycle threshold **(Ct)** values in patients infected with DENV-1, DENV-2, or CHIKV. **(D)** Comparison of Ct values between male and female patients within each infection group. **(E–G)** Correlation analyses between age and Ct values in patients infected with DENV-1 **(E)**, DENV-2 **(F)**, or CHIKV **(G)**. Correlation coefficients (*r)* and corresponding *P* values are indicated. Data in **(D)** were analyzed using the Mann-Whitney U test and are presented as median with interquartile range (IQR). Data in **(E–G)** were analyzed using Spearman’s rank correlation test. A two-tailed *P* value < 0.05 was considered statistically significant.

### 3.2 Pathogen-specific antibody responses are associated with Ct values

To characterize humoral immune responses, ELISA was performed to quantify DENV-1 NS1-, DENV-2 NS1-, and CHIKV E2-specific IgM and IgG. As expected, both IgM and IgG responses were significantly elevated in all infected groups compared with healthy controls (**Figures 2A–F**). As shown in **Figures 2G–I**, Spearman’s rank correlation analysis identified significant positive associations between virus-specific IgM levels and Ct values in DENV-1 (*r* = 0.4475, BH-adjusted *P* = 0.0447), and CHIKV (*r* = 0.4414, BH-adjusted *P* = 0.0480) infections. Although the association between virus-specific IgM levels and Ct values in DENV-2 did not reach statistical significance after BH correction (BH-adjusted *P* = 0.0622), a positive correlation was observed (*r* = 0.6626). In contrast, a significant positive correlation between IgG levels and Ct values was observed only in DENV-1 infection (Spearman’s rank correlation, *r* = 0.4846, BH-adjusted *P* = 0.0447; **Figure 2J**), whereas no significant correlations were detected in DENV-2 (**Figure 2K**) or CHIKV (**Figure 2L**) infections. To further illustrate these associations, patients were stratified into high- and low-Ct value groups according to the median Ct value within each infection group. The corresponding median Ct values used for stratification are presented in **Supplementary Table 1**. Consistent with the correlation analysis, DENV-1-infected patients with lower Ct values exhibited significantly lower IgM and IgG levels (*P* < 0.05, **Figure 2M**). However, no significant differences in IgG or IgM levels between patients with different Ct values were observed in either the DENV-2 (**Figure 2N**) or CHIKV (**Figure 2O**) groups. These results remained unchanged after adjustment for multiple comparisons using the BH method (**Supplementary Table 2**). Sex-stratified analyses showed no significant differences in IgM and IgG antibody levels between females and males across the three viral infections (**Figures 2P–R**). Together, these findings support an association between pathogen-specific antibody responses, particularly IgM responses, and Ct values in DENV-1 and CHIKV infections, while the corresponding association observed in DENV-2 did not remain statistically significant after multiple-testing correction.

**Figure 2:**
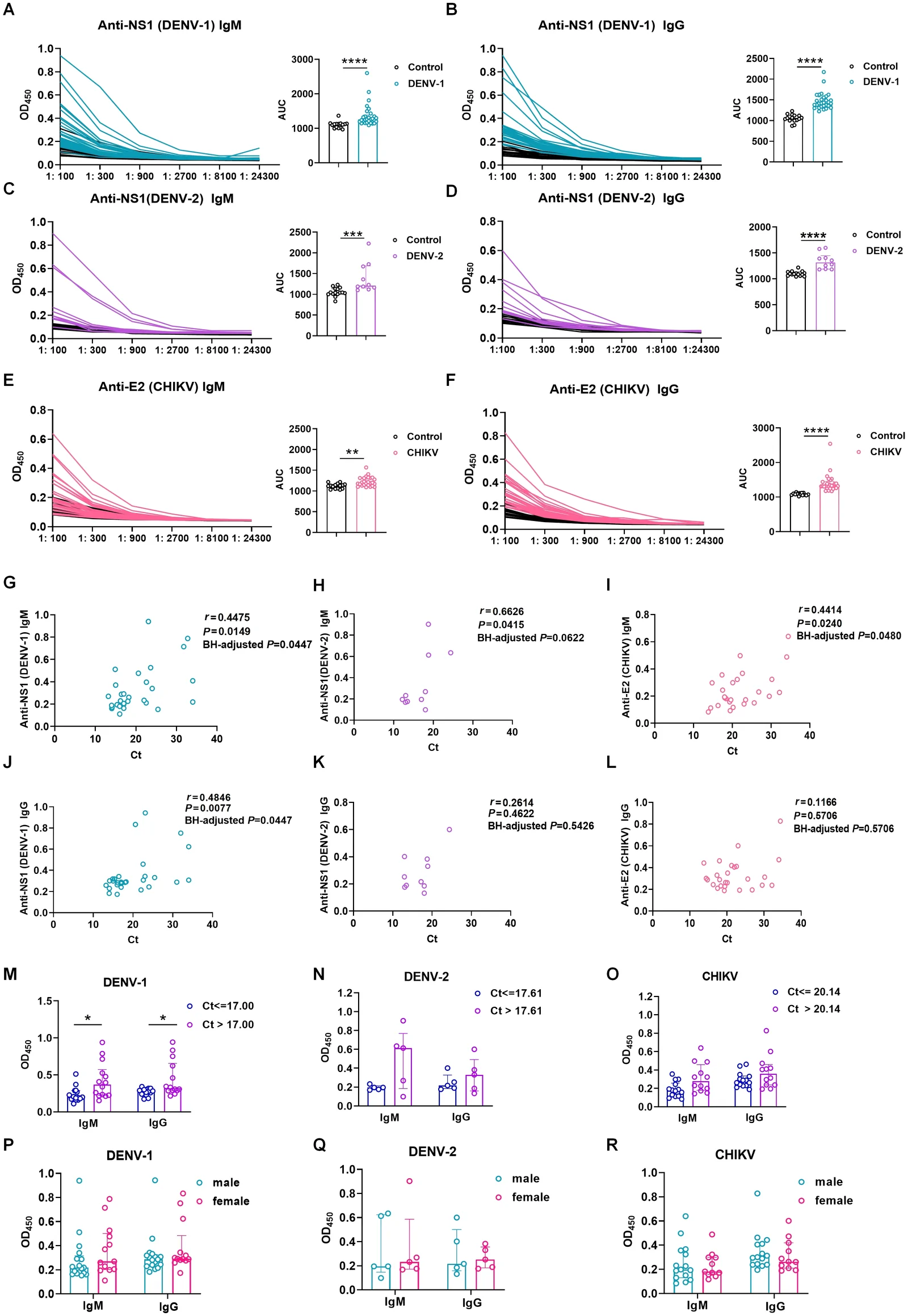
Virus-specific antibody responses and their associations with Ct values and sex in patients with DENV-1, DENV-2, and CHIKV infections. **(A–F)** Comparison of virus-specific IgG and IgM antibody levels between healthy controls and patients infected with DENV-1 **(A, B)**, DENV-2 **(C, D)**, or CHIKV **(E, F)**. Antibody levels were measured by indirect ELISA and expressed as area under the curve (AUC). Antibody levels in subsequent analyses were represented by OD values measured at a 1:100 serum dilution. **(G–L)** Correlation analyses between virus-specific IgG or IgM antibody levels and Ct values in patients infected with DENV-1 **(G, J)**, DENV-2 **(H, K)**, or CHIKV **(I, L)**. **(M–O)** Comparison of virus-specific IgG and IgM antibody levels between low-Ct and high-Ct groups in patients infected with DENV-1 **(M)**, DENV-2 **(N)**, or CHIKV **(O)**. **(P–R)** Comparison of virus-specific IgM and IgG antibody levels between male and female patients infected with DENV-1 **(P)**, DENV-2 **(Q)**, or CHIKV **(R)**. Data in **(A–F, M–R)** are presented as median with interquartile range (IQR) and were analyzed using the Mann-Whitney U test. Data in **(G-L)** were analyzed using Spearman’s rank correlation test. For each antibody-related analytical purpose, multiple-testing correction was performed across the three infection groups and two antibody measurements (IgG and IgM), resulting in six tests per BH correction. *P* values were adjusted using the BH procedure. Spearman’s correlation coefficients (*r*) and corresponding BH-adjusted P values are shown. A two-tailed BH-adjusted *P* < 0.05 was considered statistically significant. *P < 0.05, **P < 0.01, ***P < 0.001, ****P < 0.0001.

### 3.3 DENV-1, DENV-2, and CHIKV infections exhibit shared and virus-associated immune signatures

To characterize inflammatory responses induced by DENV-1, DENV-2, and CHIKV infection, serum cytokine and chemokine levels were quantified using a Bio-Plex multiplex assay. Compared with healthy controls, all three viral infections exhibited a broadly similar inflammatory profile, characterized by significantly reduced levels of the pro-inflammatory cytokine MIF, the chemokines CCL3, CCL7 and CXCL8, as well as the growth factor G-CSF (**Figures 3A–E**). In contrast, TRAIL and CXCL10 were consistently elevated in all three infection groups (**Figures 3F, G**). Additional immune alterations were shared between subsets of viral infections. IL-10 was significantly increased in both DENV-1- and DENV-2-infected patients (**Figure 3H**), whereas CXCL9 was elevated in DENV-1- and CHIKV-infected patients (**Figure 3I**). Additionally, CCL4, CCL5 and TNF-α were significantly reduced in patients with DENV-1 and DENV-2 infection but remained comparable to healthy controls in the CHIKV-infected group (**Figures 3J–L**). Distinct virus-associated immune signatures were also observed. DENV-1 infection was uniquely associated with increased IL-1Ra (**Figure 3M**). DENV-2 infection was characterized by selective reductions in PDGF-BB, HGF, IL-9 and bFGF, together with an elevated level of M-CSF (**Figures 3N–R**). In contrast, CHIKV infection uniquely exhibited increased IL-1α levels compared with healthy controls (**Figure 3S**). To assess the potential influence of sex imbalance, sensitivity analyses were repeated among male participants only. In the male-only analyses, some of the cytokine differences identified in the overall analyses were no longer statistically significant, including differences in TRAIL, CCL5, TNF-α, IL-1Ra, and IL-10 in DENV-1 infection; IL-10, TNF-α, TRAIL, CCL5, M-CSF, and IL-9 in DENV-2 infection; and CCL3, TRAIL, G-CSF, CXCL9, and MIF in CHIKV infection (**Supplementary Tables 3**–**5**). Collectively, these findings indicate that although DENV-1, DENV-2, and CHIKV infections share a common pattern of inflammatory suppression, each virus elicits a distinct immune signature, highlighting differential host immune responses to these arboviral infections.

**Figure 3:**
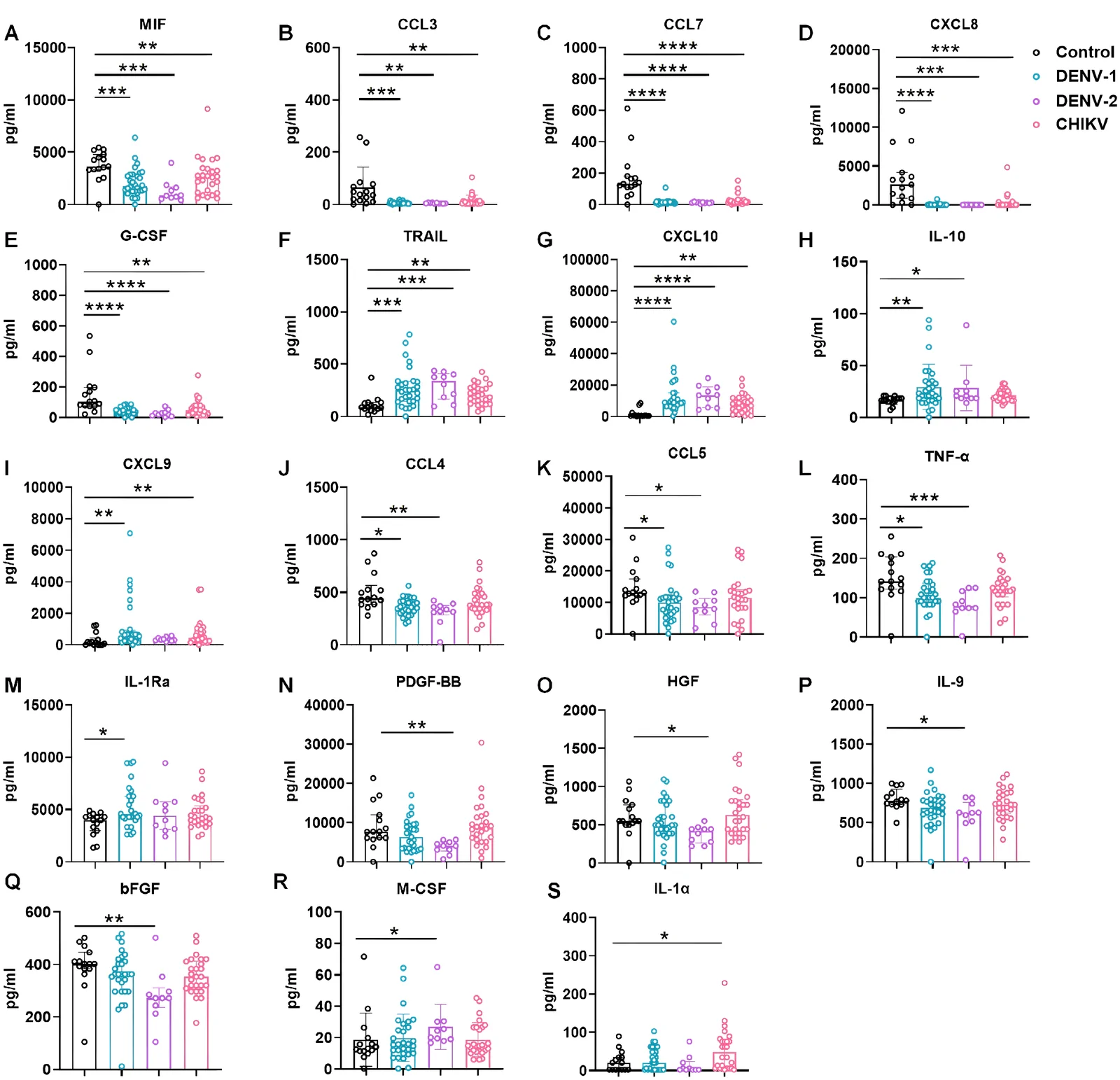
Altered serum cytokine profiles in DENV-1-, DENV-2-, and CHIKV-infected patients. **(A–S)** Comparison of serum cytokine concentrations between healthy controls and patients infected with DENV-1, DENV-2, or CHIKV. Serum cytokine levels were quantified using a Bio-Plex multiplex assay. Data in **(A–S)** were analyzed using the Kruskal-Wallis test, followed by Dunn’s multiple comparisons test with Bonferroni correction for multiple comparisons, and are presented as median with interquartile range (IQR). A two-tailed *P* value < 0.05 was considered statistically significant. *P < 0.05, **P < 0.01, ***P < 0.001, ****P < 0.0001.

### 3.4 Distinct cytokine-antibody association patterns across viral infections

To explore immune coordination, Spearman’s correlation analyses were performed to evaluate cytokine-antibody and cytokine-cytokine associations within each infection group. Cytokine-antibody correlations showed distinct patterns across the three infections. In DENV-1 infection, both IgM and IgG exhibited broadly inverse correlations with cytokines, with shared negative associations involving bFGF, HGF, IL-2, IL-4, IL-7, and TNF-α. IgM additionally showed negative correlations with IL-1α, CXCL8, IL-16, IL-17A, MIF, and CXCL12, whereas IgG showed additional inverse correlations with G-CSF and CCL2 (**Figure 4A**). DENV-2-specific IgG displayed a more restricted pattern, with negative correlations involving CXCL1, IL-1β, IL-1Ra, IL-9, IL-12p40, CCL4, TNF-β, and TRAIL (**Figure 4B**). In CHIKV infection, IgM showed positive correlations with IL-1β, IL-2, and IL-4, whereas no significant cytokine-IgG correlations were observed (**Figure 4C**). However, none of these cytokine-antibody associations remained significant after BH correction (**Supplementary Tables 6**–**11**), and they were therefore considered exploratory. In addition, cytokine-cytokine correlation analysis revealed infection-dependent patterns of immune mediator co-variation. After BH correction, 214, 27, and 295 significant positive correlations were retained in the DENV-1, DENV-2, and CHIKV groups, respectively, with no significant negative correlations. The Venn analysis revealed 121 pairs shared between DENV-1 and CHIKV, DENV-2 shared 15 pairs with DENV-1 and 19 pairs with CHIKV (**Figure 4D**). Twelve correlation pairs were shared across all three groups, including several associations involving IL-9, TNF-β, CCL4, and CXCL1, such as IL-9-TNF-β, CCL4-IL-9, CCL4-TNF-β, CXCL1-IL-9, CCL4-CXCL1, and CXCL1-TNF-β (**Figure 4E**). These findings provide insights into both shared and infection-associated patterns of immune mediator coordination across the three arboviral infections.

**Figure 4:**
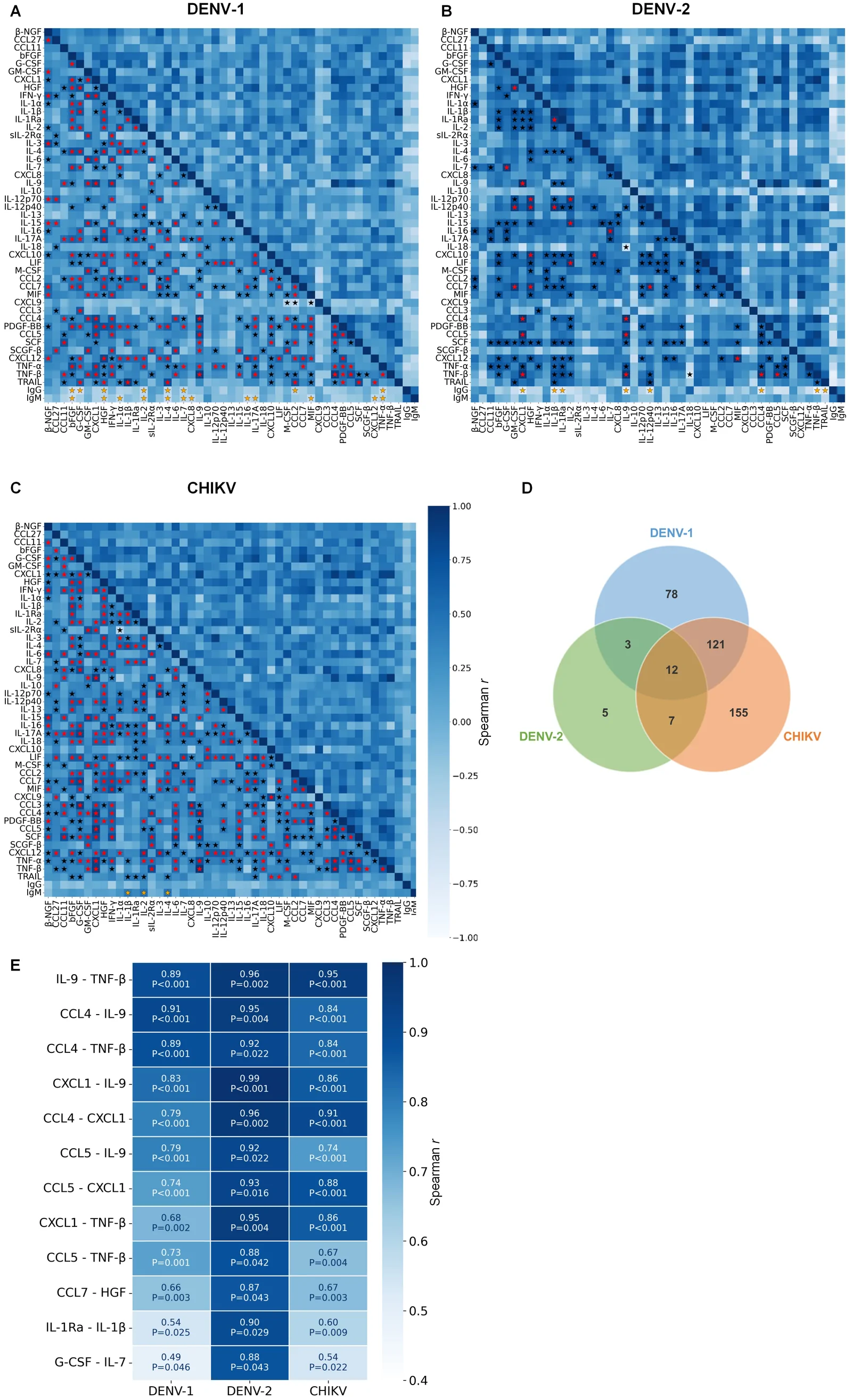
Correlation patterns among cytokines and between cytokines and virus-specific antibody responses in DENV-1, DENV-2, and CHIKV infections. **(A–C)** Heatmaps showing Spearman correlation analyses among serum cytokine concentrations and between cytokine levels and virus-specific IgM and IgG antibody responses in patients infected with DENV-1 **(A)**, DENV-2 **(B)**, or CHIKV **(C)**. Color intensity represents Spearman’s correlation coefficient (*r*). For cytokine-cytokine correlations, *P* values were adjusted for multiple comparisons using the BH procedure, with 990 pairwise comparisons included in each infection group. For cytokine-antibody correlations, *P* values were also adjusted using the BH procedure, with 90 tests included in each infection group. Black asterisks indicate cytokine-cytokine correlations that were nominally significant before multiple-testing correction (*P* < 0.05), yellow asterisks indicate cytokine-antibody correlations that were nominally significant before multiple-testing correction (*P* < 0.05), and red asterisks indicate correlations that remained significant after BH correction (BH-adjusted *P* < 0.05). **(D)** Venn diagram showing the overlap of BH-adjusted significant cytokine-cytokine correlation pairs among the three infection groups. **(E)** Heatmap of the 12 cytokine-cytokine correlation pairs shared across DENV-1, DENV-2, and CHIKV. Cell colors and numbers indicate Spearman’s correlation coefficients (*r*), with BH-adjusted *P* values shown below.

### 3.5 Inflammatory responses are differentially associated with Ct value and sex

Associations of inflammatory responses with Ct values and sex were further evaluated. In DENV-1-infected patients, correlation analysis revealed that bFGF, G-CSF, HGF, IL-1β, IL-4, CCL2, SCF and TRAIL were negatively correlated with Ct values, whereas IL-10 was positively correlated with Ct values (**Figure 5A**). However, no significant correlations were observed in DENV-2-infected (**Figure 5B**) and CHIKV-infected patients (**Figure 5C**). To further explore these associations, high- and low-Ct groups were compared within each infection group. In DENV-1-infected patients, the low-Ct group showed significantly elevated IL-4, G-CSF and IL-7, whereas IL-10 level was significantly reduced (**Figure 5D**). No significant differences in cytokine levels were observed between the two Ct subgroups in DENV-2-infected (**Supplementary Figures 1E–H**) and CHIKV-infected patients (**Supplementary Figures 1I–L**). Sex-stratified analysis revealed higher levels of MIF, PDGF-BB, and CXCL12 in male than in female patients with DENV-2 infection (**Figure 5E**). In CHIKV-infected patients, females exhibited higher levels of IL-9, SCF, and TNF-β, whereas sIL-2Rα levels were higher in males than in females (**Figure 5F**). In DENV-1-infected patients, sex had no significant impact on cytokines (**Supplementary Figures 2A–D**). Following BH correction for multiple comparisons, the negative correlations of IL-1β, IL-4, and TRAIL with Ct values in DENV-1-infected patients remained statistically significant (**Figure 5A**; **Supplementary Table 12**). In contrast, among the high- versus low-Ct group comparisons, IL-4 was the only cytokine that remained statistically significant after correction (**Figure 5D**; **Supplementary Table 13**). None of the observed sex-associated differences in cytokine levels remained statistically significant after multiple-testing correction (**Supplementary Tables 14**, **15**). Collectively, these results suggest distinct patterns of inflammatory responses associated with Ct values and sex across DENV-1, DENV-2, and CHIKV infections, with DENV-1 exhibiting the most pronounced Ct value-associated cytokine variation.

**Figure 5:**
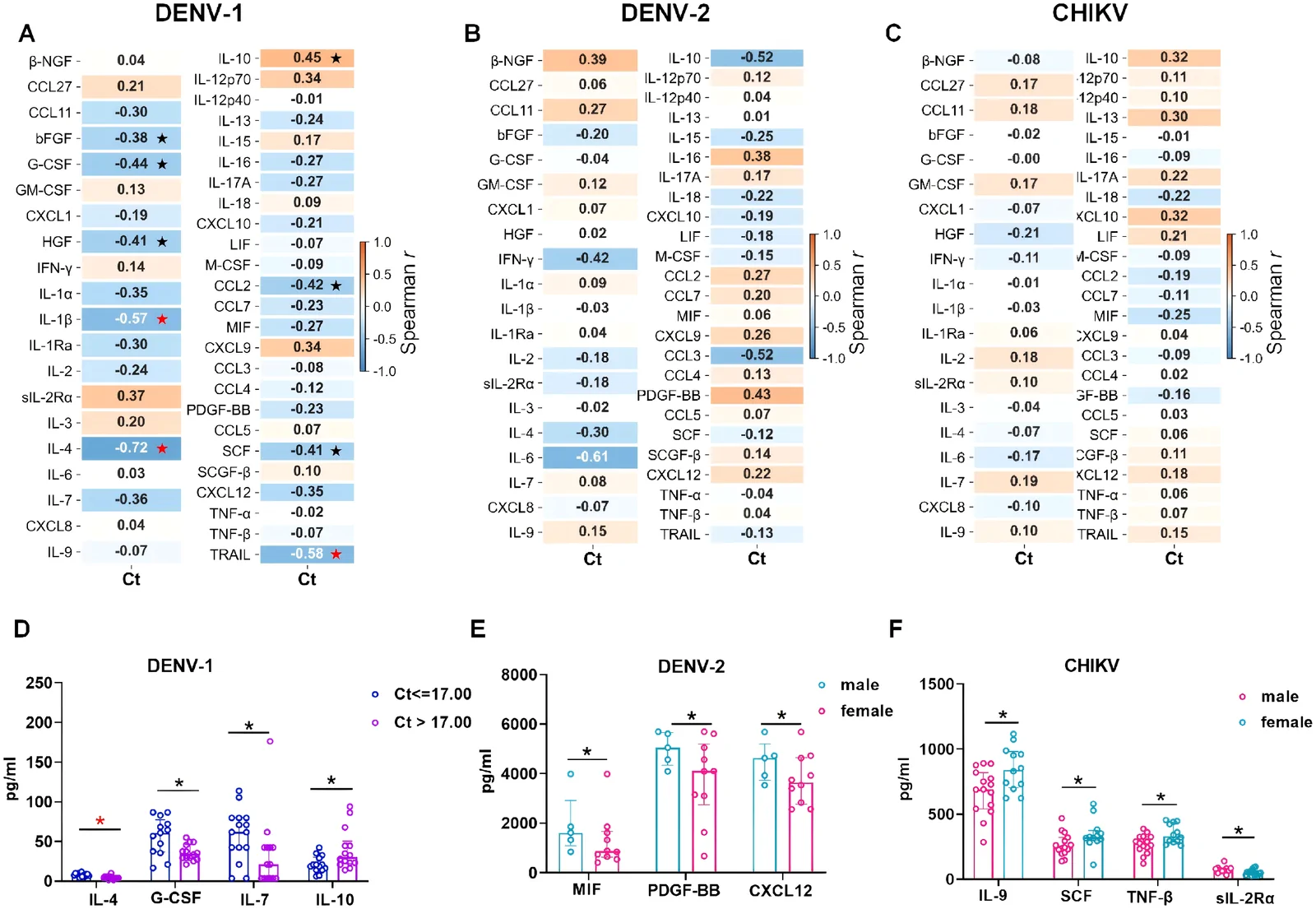
Associations of cytokine responses with Ct values and sex. **(A–C)** Heatmaps showing Spearman correlation analyses between serum cytokine concentrations and Ct values in patients infected with DENV-1 **(A)**, DENV-2 **(B)**, or CHIKV **(C)**. Color intensity represents Spearman’s correlation coefficient (*r*). **(D)** Comparison of serum cytokine levels in DENV-1 patients stratified by Ct values. **(E, F)** Comparison of serum cytokine levels between male and female patients infected with DENV-2 **(E)** or CHIKV **(F)**. Data in **(A–C)** were analyzed using Spearman’s rank correlation test. Data in **(D–F)** were analyzed using the Mann-Whitney U test and are presented as median with interquartile range (IQR). For each analysis in **(A–F)**, 45 cytokine tests were included in the BH correction. Black asterisks indicate nominally significant associations before multiple-comparison correction (unadjusted *P* < 0.05), whereas red asterisks indicate associations that remained statistically significant after BH correction (BH-adjusted *P* < 0.05).

## 4 Discussion

This study provides a comparative characterization of host immune responses during acute DENV-1, DENV-2, and CHIKV infections. Despite belonging to distinct viral lineages, these infections shared several features of humoral and inflammatory responses while exhibiting distinct patterns of immune regulation. By integrating viral RNA levels, pathogen-specific antibody responses, cytokine profiles, and pairwise correlation patterns, our study extends previous comparative analyses (28) by providing an integrated assessment of the relationships among viral burden, humoral immunity, and systemic inflammation. This approach revealed distinct patterns of immune coordination across DENV-1, DENV-2, and CHIKV infections, highlighting differential interactions between pathogen burden, antibody responses, and inflammatory mediators.

One of the most striking observations was that all three infections shared a conserved inflammatory framework characterized by elevated CXCL10 and TRAIL together with restrained systemic inflammation. Since both mediators are frequently induced downstream of interferon signaling during acute viral infection (30–33), their consistent elevation may indicate convergence of early antiviral immune activation across *Flavivirus* and *Alphavirus* infections, despite their distinct virological characteristics. Notably, CXCL10, TRAIL, and IL-6 have also been reported during other viral infections, including influenza and SARS-CoV-2 infections (34, 35), indicating that these individual mediators are not specific to DENV or CHIKV but rather reflect shared antiviral and inflammatory pathways. Nevertheless, different viruses can induce distinct combinations, magnitudes, and temporal dynamics of immune responses (28, 36). Thus, although individual inflammatory mediators are not virus-specific, their coordinated patterns, together with pathogen RNA levels and antibody responses, may better capture the coordinated nature of infection-associated immune responses. In contrast, classical pro-inflammatory cytokines associated with severe arboviral disease, such as IL-6, TNF-α, and IL-1β, were not broadly elevated in our cohort (14, 15, 37). This pattern may be influenced by differences in infection stage or clinical context; however, these factors could not be evaluated because relevant clinical and temporal data were not systematically collected. Therefore, the limited elevation of these cytokines should be interpreted cautiously and should not be considered evidence that severity-associated inflammatory pathways are absent during arboviral infections. Overall, our findings suggest a shared pattern of interferon-associated antiviral activation across the three infections, accompanied by limited systemic pro-inflammatory responses in this cohort.

Despite this shared antiviral immune framework, each arbovirus established a distinct immune landscape, indicating that downstream immune regulation diverges following a common early antiviral response. DENV-1 was characterized by increased CXCL9 together with the regulatory mediators IL-10 and IL-1Ra, consistent with concurrent interferon-associated and regulatory immune responses (38, 39). By contrast, DENV-2 exhibited broader alterations in inflammatory mediators and growth factors, including reduced PDGF-BB, HGF, and bFGF, accompanied by increased M-CSF. These changes are consistent with altered regulation of monocyte/macrophage-associated and tissue-repair-related pathways (40, 41), although their functional significance remains to be determined. Compared with DENV-1 and DENV-2, CHIKV infection showed a more restricted cytokine signature, with elevated IL-1α. While prior studies of acute CHIKV have largely focused on increased IL-6, IFN-γ, and IL-17, IL-1α elevation has not been consistently reported (42–44). Our observation of increased IL-1α may thus represent a distinct feature of the host response requiring validation. Together, these observations suggest that although DENV-1, DENV-2, and CHIKV activate a conserved antiviral program, the downstream organization of host immunity differs substantially according to viral biology, tissue tropism, and the balance between inflammatory and regulatory pathways.

Beyond differences in circulating immune mediators, pairwise correlation analysis suggested differences in the patterns of associations among the three arboviral infections. Positive associations between pathogen-specific IgM and Ct values suggested a relationship between humoral responses and viral RNA levels, although the association was not statistically significant for DENV-2 after multiple-testing correction. This finding suggests that IgM responses may represent a common component of host defense during acute arboviral infection. The integration of viral RNA with inflammatory and humoral responses nevertheless differed among the three infections, with DENV-1 showing a broader pattern of statistically supported associations than DENV-2. This difference should be interpreted cautiously given the smaller DENV-2 sample size and the lack of information on previous DENV exposure and primary versus secondary infection status. CHIKV showed an apparent pattern of positive associations between inflammatory cytokines and virus-specific IgM responses. Previous studies have suggested that persistent viral antigen and prolonged immune stimulation may contribute to sustained inflammatory and humoral responses during CHIKV infection (45–47). Besides, cytokine-cytokine correlation analysis revealed both shared and infection-specific patterns of immune mediator co-variation. The shared associations centered on IL-9, TNF-β, CCL4, and CXCL1, which have been implicated in inflammatory and leukocyte-recruiting responses during DENV and CHIKV (43, 48, 49). Their coordinated variation may therefore represent a common feature of acute arboviral immune responses. However, these correlations do not establish direct regulatory interactions or shared upstream mechanisms. Collectively, these findings suggest that arboviruses share several common immune response features but differ in the relationships among viral RNA levels, inflammatory responses, and humoral immunity, highlighting distinct host immune response patterns during acute infection.

This study has several limitations. First, its cross-sectional design and lack of systematically recorded infection-stage and clinical information limited assessment of the temporal and clinical relevance of the observed immune responses, including IgM-Ct associations. Second, previous DENV exposure and primary versus secondary infection status were not systematically assessed. In addition, DENV-CHIKV coinfection, previous heterologous arbovirus exposure, and potential cross-reactive antibody responses were not specifically evaluated. Third, the relatively small DENV-2 group (n = 10) limited statistical power; therefore, DENV-2 stratified and correlation findings should be considered exploratory. In addition, differences in sample size between infection groups may influence the number of detected associations, and comparisons of immune interaction patterns between DENV-1 and DENV-2 should therefore be interpreted cautiously. Fourth, although some cytokine-cytokine associations remained significant after BH correction, most nominally significant cytokine-antibody and cytokine-Ct associations did not remain significant after correction. These findings underscore the need for cautious interpretation of nominal associations and validation in larger cohorts. Fifth, the absence of broader viral comparison groups limits assessment of the generalizability of the identified immune-response patterns. Future studies comparing additional viral infections, including influenza and SARS-CoV-2, will be required to determine whether these immune-response patterns represent shared antiviral mechanisms or infection-specific features. Finally, residual demographic confounding cannot be excluded, particularly given differences in age and sex distributions between infected participants and healthy controls.

In summary, DENV-1, DENV-2, and CHIKV shared several inflammatory features but exhibited distinct patterns of immune associations. DENV-1 showed a broader pattern of statistically supported associations among viral RNA levels, cytokines, and antibody responses, whereas CHIKV displayed a distinct cytokine-antibody association pattern. Together, these findings provide an integrated comparative view of shared and virus-associated immune responses during acute arboviral infection and extend previous cytokine-focused comparisons by linking inflammatory, humoral, and viral RNA profiles.

## Data Availability

The original contributions presented in the study are included in the article/**Supplementary Material**. Further inquiries can be directed to the corresponding authors.
