# Supplementary material for: Comparative analysis of serum antibody responses and immune landscapes in patients with acute DENV-1, DENV-2, and Chikungunya virus infections
Source: Front Immunol. 2026 Oct 28;17:1948489. doi: 10.3389/fimmu.2026.1948489 (PMC13752185; doi:10.3389/fimmu.2026.1948489)
Supplement: Supplementary file 1 [file DataSheet1.docx]

**Supplementary Material**

**Figure S1. Cytokines not significantly associated with Ct values in DENV-1-, DENV-2- and CHIKV-infected patients**

(A-L) Comparison of cytokine levels in patients infected with DENV-1 (A-D), DENV-2 (E-H), or CHIKV (I-L) stratified by Ct value groups. Data in (A-L) were analyzed using the Mann-Whitney U test and are presented as median with interquartile range (IQR). A two-tailed *P* value < 0.05 was considered statistically significant. The reported *P* values were not adjusted for multiple comparisons.

**Figure S2. Cytokines not significantly associated with sex in DENV-1-, DENV-2- and CHIKV-infected patients**

(A-L) Comparison of cytokine levels between male and female patients infected with DENV-1 (A-D), DENV-2 (E-H) or CHIKV (I-L). Data in (A-L) were analyzed using the Mann-Whitney U test and are presented as median with interquartile range (IQR). A two-tailed *P* value < 0.05 was considered statistically significant. The reported *P* values were not adjusted for multiple comparisons.

**Table S1.** Ct value cut-offs used for stratification of patients into high- and low-Ct groups

| Infection group | Median Ct value (cut-off) | Low-Ct group | High-Ct group |
| --- | --- | --- | --- |
| DENV-1 | 17.00 | ≤17.00 | >17.00 |
| DENV-2 | 17.61 | ≤17.61 | >17.61 |
| CHIKV | 20.14 | ≤20.14 | >20.14 |

Patients within each infection group were stratified into low- and high-Ct groups according to the median Ct value of the respective group.

| Virus | Antibody | U statistic | HL (95% CI) | Raw *P* | BH-adjusted *P* |
| --- | --- | --- | --- | --- | --- |
| DENV-1 | IgM | 47 | -0.122 (-0.297, -0.031) | 0.012 | 0.042 |
|  | IgG | 48 | -0.062 (-0.341, -0.013) | 0.014 | 0.042 |
| DENV-2 | IgM | 5 | -0.418 (-0.671, 0.070) | 0.144 | 0.199 |
|  | IgG | 11 | -0.078 (-0.348, 0.072) | 0.835 | 0.835 |
| CHIKV | IgM | 39 | -0.069 (-0.193, 0.007) | 0.073 | 0.146 |
|  | IgG | 61 | -0.068 (-0.171, 0.023) | 0.166 | 0.199 |

**Table S2.** Comparison of virus-specific IgM and IgG antibody levels among different Ct value groups in patients infected with DENV-1, DENV-2, or CHIKV.

Patients were divided into low-Ct and high-Ct groups according to the median Ct value. Virus-specific IgM and IgG levels were compared using the Mann-Whitney U test. U statistics, Hodges-Lehmann (HL) estimates (low Ct - high Ct) with 95% confidence intervals (CIs), raw *P* values, and BH-adjusted *P* values are shown. For multiple-testing correction, six tests were included in the BH correction. BH-adjusted *P* < 0.05 was considered statistically significant.

| Cytokine | U statistic | HL (95% CI) | Adjusted *P* |
| --- | --- | --- | --- |
| CXCL10 | 161.000 | 8373.480 (6188.297, 11716.002) | <0.001 |
| CCL7 | 13.000 | -147.800 (-172.440, -101.660) | 0.001 |
| MIF | 17.000 | -2248.780 (-2914.470, -1115.570) | 0.002 |
| CXCL8 | 21.000 | -3194.520 (-4165.633, -796.549) | 0.003 |
| G-CSF | 26.000 | -78.780 (-143.070, -18.290) | 0.009 |
| CCL3 | 28.000 | -39.660 (-72.170, -7.690) | 0.012 |
| CXCL9 | 125.500 | 604.410 (139.399, 2261.551) | 0.031 |
| CCL4 | 36.000 | -114.780 (-232.390, -32.760) | 0.042 |
| TRAIL | 131.000 | 169.700 (20.500, 344.690) | 0.055 |
| CCL5 | 39.000 | -4464.230 (-6863.690, -1053.220) | 0.063 |
| TNF-α | 40.500 | -33.660 (-69.690, -8.250) | 0.076 |
| IL-1Ra | 114.500 | 1266.720 (0.000, 2841.070) | 0.147 |
| IL-10 | 117.500 | 6.920 (-1.960, 16.830) | 0.282 |

**Table S3.** Sex-restricted sensitivity analysis of serum cytokine levels in male patients with acute DENV-1 infection versus male healthy controls.

Only male participants were included in this sensitivity analysis to assess the potential influence of sex imbalance between infected patients and healthy controls. Cytokine levels were compared among the DENV-1, DENV-2, CHIKV, and healthy control groups using the Kruskal-Wallis test followed by Dunn’s post-hoc test with Bonferroni correction. U, Mann-Whitney U statistic; HL, Hodges-Lehmann estimate of the location shift between infected patients and healthy controls; 95% CI, 95% confidence interval. The reported *P* values are Bonferroni-adjusted, and a Bonferroni-adjusted *P* < 0.05 was considered statistically significant.

**Table S4.** Sex-restricted sensitivity analysis of serum cytokine levels in male patients with acute DENV-2 infection versus male healthy controls.

| Cytokine | U statistic | HL (95% CI) | Adjusted *P* |
| --- | --- | --- | --- |
| G-CSF | 1.500 | -78.780 (-182.870, -53.640) | 0.008 |
| CXCL10 | 61.000 | 5322.860 (4975.840, 18915.510) | 0.008 |
| CCL7 | 5.000 | -126.690 (-178.440, -99.700) | 0.013 |
| MIF | 5.000 | -3000.080 (-3931.920, -2666.200) | 0.013 |
| bFGF | 4.500 | -170.170 (-278.510, -105.030) | 0.020 |
| CCL4 | 6.000 | -207.630 (-410.280, -67.957) | 0.020 |
| PDGF-BB | 6.000 | -5441.330 (-11809.700, -2963.780) | 0.031 |
| CXCL8 | 7.000 | -3247.590 (-4165.633, -822.300) | 0.038 |
| HGF | 7.000 | -329.740 (-488.200, -80.510) | 0.040 |
| CCL3 | 8.000 | -31.920 (-74.080, -6.790) | 0.042 |
| IL-10 | 57.000 | 15.290 (2.310, 68.050) | 0.052 |
| TNF-α | 8.000 | -84.620 (-137.220, -24.240) | 0.053 |
| TRAIL | 57.000 | 111.670 (25.840, 328.680) | 0.054 |
| CCL5 | 12.000 | -7917.540 (-11255.470, -524.210) | 0.138 |
| M-CSF | 52.000 | 9.750 (2.970, 48.880) | 0.183 |
| IL-9 | 17.000 | -188.100 (-680.580, 35.710) | 0.417 |

Male-only sensitivity analysis. Cytokine levels were compared using the Kruskal-Wallis test followed by Dunn’s post-hoc test with Bonferroni correction. U, Mann-Whitney U statistic; HL, Hodges-Lehmann estimate of the location shift between infected patients and healthy controls; 95% CI, 95% confidence interval. *P* values are Bonferroni-adjusted; *P* < 0.05 was considered statistically significant.

**Table S5.** Sex-restricted sensitivity analysis of serum cytokine levels in male patients with acute CHIKV infection versus male healthy controls.

| Cytokine | U statistic | HL (95% CI) | Adjusted *P* |
| --- | --- | --- | --- |
| CCL7 | 17.000 | -116.360 (-168.330, -92.240) | 0.001 |
| CXCL10 | 169.000 | 4841.550 (2976.550, 8551.960) | 0.003 |
| CXCL8 | 32.000 | -2245.500 (-4165.633, -298.043) | 0.008 |
| IL-1α | 151.500 | 40.280 (3.701, 73.671) | 0.039 |
| CCL3 | 47.500 | -30.760 (-65.980, -1.900) | 0.068 |
| TRAIL | 147.000 | 74.790 (13.850, 192.300) | 0.072 |
| G-CSF | 53.500 | -56.810 (-130.840, -3.670) | 0.135 |
| CXCL9 | 130.500 | 236.130 (-30.060, 583.439) | 0.151 |
| MIF | 56.000 | -1085.120 (-2480.710, 4.940) | 0.177 |

Male-only sensitivity analysis. Cytokine levels were compared using the Kruskal-Wallis test followed by Dunn’s post-hoc test with Bonferroni correction. U, Mann-Whitney U statistic; HL, Hodges-Lehmann estimate of the location shift between infected patients and healthy controls; 95% CI, 95% confidence interval. *P* values are Bonferroni-adjusted; *P* < 0.05 was considered statistically significant.

**Table S6.** Correlation analysis between serum cytokine levels and virus-specific IgM antibody levels in DENV-1-infected patients.

| Cytokine | Spearman’s r (95%CI) | Raw P | BH-adjusted P |
| --- | --- | --- | --- |
| bFGF | -0.521 (-0.763, -0.174) | 0.004 | 0.127 |
| IL-1α | -0.465 (-0.752, -0.100) | 0.011 | 0.127 |
| MIF | -0.493 (-0.741, -0.165) | 0.008 | 0.127 |
| IL-4 | -0.498 (-0.742, -0.176) | 0.008 | 0.127 |
| IL-16 | -0.474 (-0.727, -0.118) | 0.011 | 0.127 |
| CXCL12 | -0.464 (-0.779, -0.102) | 0.013 | 0.128 |
| CXCL8 | -0.435 (-0.709, -0.071) | 0.018 | 0.138 |
| IL-7 | -0.419 (-0.691, -0.037) | 0.024 | 0.163 |
| IL-2 | -0.417 (-0.706, -0.043) | 0.027 | 0.176 |
| HGF | -0.404 (-0.686, -0.057) | 0.033 | 0.186 |
| IL-17A | -0.392 (-0.687, -0.037) | 0.039 | 0.200 |
| TNF-α | -0.375 (-0.645, 0.008) | 0.049 | 0.221 |
| CCL11 | -0.367 (-0.673, 0.017) | 0.055 | 0.234 |
| CXCL1 | -0.349 (-0.681, 0.074) | 0.069 | 0.276 |
| CCL4 | -0.331 (-0.686, 0.121) | 0.086 | 0.297 |
| PDGF-BB | -0.316 (-0.689, 0.101) | 0.095 | 0.304 |
| G-CSF | -0.315 (-0.671, 0.100) | 0.110 | 0.326 |
| IL-10 | 0.278 (-0.100, 0.590) | 0.152 | 0.402 |
| IL-9 | -0.274 (-0.644, 0.171) | 0.158 | 0.406 |
| CCL7 | -0.270 (-0.565, 0.075) | 0.165 | 0.412 |
| TNF-β | -0.263 (-0.621, 0.156) | 0.176 | 0.428 |
| IFN-γ | -0.241 (-0.578, 0.160) | 0.208 | 0.467 |
| CCL2 | -0.234 (-0.599, 0.166) | 0.222 | 0.467 |
| IL-1Ra | -0.230 (-0.560, 0.160) | 0.248 | 0.485 |
| β-NGF | -0.222 (-0.534, 0.144) | 0.247 | 0.485 |
| CCL5 | -0.218 (-0.591, 0.183) | 0.255 | 0.489 |
| LIF | -0.216 (-0.571, 0.176) | 0.269 | 0.503 |
| TRAIL | -0.206 (-0.545, 0.184) | 0.292 | 0.515 |
| SCF | -0.192 (-0.614, 0.231) | 0.329 | 0.569 |
| IL-3 | -0.176 (-0.537, 0.222) | 0.362 | 0.592 |
| IL-15 | -0.165 (-0.460, 0.153) | 0.392 | 0.625 |
| IL-6 | -0.167 (-0.555, 0.257) | 0.396 | 0.625 |
| CXCL10 | -0.135 (-0.514, 0.245) | 0.485 | 0.693 |
| CXCL9 | 0.130 (-0.294, 0.527) | 0.520 | 0.719 |
| sIL-2Rα | 0.122 (-0.247, 0.449) | 0.536 | 0.720 |
| IL-12p70 | -0.120 (-0.498, 0.280) | 0.544 | 0.720 |
| IL-1β | -0.112 (-0.493, 0.284) | 0.570 | 0.743 |
| IL-12p40 | -0.106 (-0.488, 0.289) | 0.590 | 0.748 |
| IL-13 | -0.081 (-0.488, 0.370) | 0.684 | 0.813 |
| CCL27 | -0.074 (-0.470, 0.345) | 0.701 | 0.820 |
| IL-18 | 0.062 (-0.347, 0.483) | 0.755 | 0.853 |

**Table S6 (Continued).** Correlation analysis between serum cytokine levels and virus-specific IgM antibody levels in DENV-1-infected patients

| Cytokine | Spearman’s r (95%CI) | Raw P | BH-adjusted P |
| --- | --- | --- | --- |
| CCL3 | -0.054 (-0.426, 0.315) | 0.785 | 0.862 |
| M-CSF | 0.048 (-0.392, 0.472) | 0.809 | 0.866 |
| SCGF-β | -0.030 (-0.443, 0.413) | 0.881 | 0.901 |
| GM-CSF | -0.022 (-0.420, 0.377) | 0.913 | 0.913 |

Spearman’s rank correlation coefficients (*r*) with 95% confidence intervals (CIs) were used to assess associations between the 45 cytokines included in the analysis and IgG and IgM antibody levels. Raw *P* values and Benjamini-Hochberg (BH)-adjusted *P* values are shown. For multiple-testing correction, cytokine- antibody correlations were adjusted separately within each infection group, with 90 tests included in each BH correction. Antibody levels were represented by OD values measured at a 1:100 serum dilution. BH-adjusted *P* < 0.05 was considered statistically significant.

**Table S7.** Correlation analysis between serum cytokine levels and virus-specific IgG antibody levels in DENV-1-infected patients.

| Cytokine | Spearman’s r (95%CI) | Raw P | BH-adjusted P |
| --- | --- | --- | --- |
| TNF-α | -0.472 (-0.705, -0.138) | 0.011 | 0.127 |
| bFGF | -0.523 (-0.751, -0.181) | 0.004 | 0.127 |
| G-CSF | -0.568 (-0.732, -0.284) | 0.002 | 0.127 |
| IL-4 | -0.451 (-0.724, -0.102) | 0.018 | 0.138 |
| IL-2 | -0.450 (-0.755, -0.041) | 0.016 | 0.138 |
| IL-7 | -0.402 (-0.705, -0.017) | 0.030 | 0.183 |
| CCL2 | -0.383 (-0.688, -0.008) | 0.040 | 0.200 |
| HGF | -0.381 (-0.685, -0.002) | 0.046 | 0.216 |
| IL-1α | -0.341 (-0.670, 0.085) | 0.071 | 0.276 |
| IL-12p40 | -0.335 (-0.691, 0.108) | 0.082 | 0.293 |
| CXCL1 | -0.336 (-0.688, 0.090) | 0.080 | 0.293 |
| IL-16 | -0.326 (-0.639, 0.079) | 0.091 | 0.303 |
| IL-1β | -0.307 (-0.616, 0.036) | 0.112 | 0.326 |
| CCL3 | -0.304 (-0.583, 0.068) | 0.116 | 0.326 |
| CCL7 | -0.305 (-0.567, 0.037) | 0.114 | 0.326 |
| IL-17A | -0.285 (-0.621, 0.140) | 0.142 | 0.388 |
| CCL4 | -0.258 (-0.615, 0.158) | 0.185 | 0.439 |
| LIF | -0.239 (-0.616, 0.182) | 0.221 | 0.467 |
| TNF-β | -0.238 (-0.587, 0.141) | 0.223 | 0.467 |
| TRAIL | -0.241 (-0.602, 0.155) | 0.217 | 0.467 |
| CCL11 | -0.231 (-0.611, 0.171) | 0.237 | 0.485 |
| CXCL12 | -0.214 (-0.573, 0.179) | 0.274 | 0.503 |
| PDGF-BB | -0.205 (-0.553, 0.179) | 0.285 | 0.513 |
| sIL-2Rα | 0.187 (-0.191, 0.518) | 0.340 | 0.577 |
| IL-1Ra | -0.188 (-0.527, 0.213) | 0.347 | 0.579 |
| IL-15 | -0.158 (-0.517, 0.262) | 0.413 | 0.641 |
| CXCL8 | -0.151 (-0.457, 0.231) | 0.434 | 0.663 |

**Table S7 (Continued).** Correlation analysis between serum cytokine levels and virus-specific IgG antibody levels in DENV-1-infected patients.

| Cytokine | Spearman’s r (95%CI) | Raw P | BH-adjusted P |
| --- | --- | --- | --- |
| MIF | -0.147 (-0.533, 0.268) | 0.455 | 0.680 |
| IL-9 | -0.145 (-0.508, 0.257) | 0.461 | 0.680 |
| IL-6 | -0.139 (-0.546, 0.277) | 0.482 | 0.693 |
| SCF | -0.127 (-0.551, 0.281) | 0.521 | 0.719 |
| IL-18 | 0.125 (-0.310, 0.532) | 0.528 | 0.719 |
| IL-12p70 | -0.108 (-0.489, 0.289) | 0.584 | 0.748 |
| CXCL9 | 0.102 (-0.303, 0.475) | 0.614 | 0.757 |
| β-NGF | 0.099 (-0.313, 0.486) | 0.608 | 0.757 |
| IL-3 | 0.087 (-0.305, 0.488) | 0.655 | 0.796 |
| CCL5 | -0.078 (-0.485, 0.315) | 0.686 | 0.813 |
| IL-10 | 0.070 (-0.299, 0.437) | 0.722 | 0.834 |
| GM-CSF | -0.060 (-0.443, 0.390) | 0.765 | 0.853 |
| IFN-γ | -0.057 (-0.421, 0.341) | 0.767 | 0.853 |
| IL-13 | -0.051 (-0.423, 0.348) | 0.795 | 0.862 |
| M-CSF | -0.039 (-0.456, 0.388) | 0.845 | 0.895 |
| SCGF-β | 0.030 (-0.408, 0.476) | 0.878 | 0.901 |
| CCL27 | 0.030 (-0.380, 0.437) | 0.876 | 0.901 |
| CXCL10 | -0.023 (-0.383, 0.366) | 0.904 | 0.913 |

Spearman’s rank correlation coefficients (*r*) with 95% confidence intervals (CIs) were used to assess associations between the 45 cytokines included in the analysis and IgG and IgM antibody levels. Raw *P* values and Benjamini-Hochberg (BH)-adjusted *P* values are shown. For multiple-testing correction, cytokine- antibody correlations were adjusted separately within each infection group, with 90 tests included in each BH correction. Antibody levels were represented by OD values measured at a 1:100 serum dilution. BH-adjusted *P* < 0.05 was considered statistically significant.

**Table S8.** Correlation analysis between serum cytokine levels and virus-specific IgM antibody levels in DENV-2-infected patients.

| Cytokine | Spearman’s r (95%CI) | Raw P | BH-adjusted P |
| --- | --- | --- | --- |
| IL-6 | -0.612 (-0.987, 0.145) | 0.060 | 0.540 |
| sIL-2Rα | -0.515 (-0.963, 0.283) | 0.128 | 0.781 |
| β-NGF | 0.354 (-0.273, 0.774) | 0.316 | 0.880 |
| CCL5 | 0.297 (-0.338, 0.788) | 0.405 | 0.880 |
| CCL2 | 0.248 (-0.482, 0.874) | 0.489 | 0.880 |
| IL-17A | 0.252 (-0.484, 0.765) | 0.483 | 0.880 |
| IL-16 | 0.261 (-0.459, 0.887) | 0.467 | 0.880 |
| IL-15 | -0.304 (-0.879, 0.436) | 0.393 | 0.880 |
| IL-13 | 0.267 (-0.597, 0.842) | 0.456 | 0.880 |
| CXCL8 | 0.281 (-0.436, 0.815) | 0.431 | 0.880 |
| IL-3 | -0.330 (-0.849, 0.424) | 0.351 | 0.880 |
| IL-2 | -0.280 (-0.950, 0.533) | 0.432 | 0.880 |
| TRAIL | -0.455 (-0.925, 0.346) | 0.187 | 0.880 |

**Table S8 (Continued).** Correlation analysis between serum cytokine levels and virus-specific IgM antibody levels in DENV-2-infected patients

| Cytokine | Spearman’s r (95%CI) | Raw P | BH-adjusted P |
| --- | --- | --- | --- |
| IL-1β | -0.336 (-0.956, 0.551) | 0.342 | 0.880 |
| IL-1α | 0.298 (-0.357, 0.808) | 0.402 | 0.880 |
| CCL27 | -0.273 (-0.888, 0.494) | 0.446 | 0.880 |
| IFN-γ | -0.291 (-0.827, 0.466) | 0.415 | 0.880 |
| CCL11 | 0.406 (-0.275, 0.862) | 0.244 | 0.880 |
| GM-CSF | -0.334 (-0.940, 0.508) | 0.345 | 0.880 |
| IL-12p70 | -0.220 (-0.814, 0.532) | 0.542 | 0.917 |
| IL-12p40 | -0.220 (-0.875, 0.547) | 0.541 | 0.917 |
| HGF | -0.215 (-0.927, 0.558) | 0.550 | 0.917 |
| SCF | -0.176 (-0.899, 0.623) | 0.627 | 0.925 |
| IL-18 | -0.176 (-0.821, 0.575) | 0.627 | 0.925 |
| IL-4 | -0.187 (-0.787, 0.704) | 0.605 | 0.925 |
| IL-9 | 0.115 (-0.585, 0.808) | 0.751 | 0.987 |
| TNF-α | 0.129 (-0.592, 0.794) | 0.722 | 0.987 |
| CXCL12 | -0.091 (-0.901, 0.694) | 0.803 | 0.987 |
| SCGF-β | 0.067 (-0.648, 0.774) | 0.855 | 0.987 |
| PDGF-BB | 0.091 (-0.753, 0.778) | 0.803 | 0.987 |
| CCL4 | 0.006 (-0.722, 0.727) | 0.987 | 0.987 |
| CCL3 | -0.122 (-0.673, 0.555) | 0.738 | 0.987 |
| CXCL9 | 0.006 (-0.765, 0.795) | 0.987 | 0.987 |
| CCL7 | -0.043 (-0.754, 0.709) | 0.907 | 0.987 |
| IL-10 | -0.019 (-0.867, 0.768) | 0.959 | 0.987 |
| IL-7 | 0.116 (-0.528, 0.737) | 0.749 | 0.987 |
| M-CSF | -0.030 (-0.752, 0.673) | 0.934 | 0.987 |
| LIF | -0.025 (-0.948, 0.680) | 0.946 | 0.987 |
| IL-1Ra | -0.061 (-0.776, 0.731) | 0.868 | 0.987 |
| bFGF | 0.006 (-0.809, 0.790) | 0.987 | 0.987 |
| G-CSF | -0.067 (-0.689, 0.603) | 0.854 | 0.987 |
| CXCL1 | 0.079 (-0.598, 0.790) | 0.829 | 0.987 |
| TNF-β | 0.030 (-0.665, 0.790) | 0.934 | 0.987 |
| MIF | -0.103 (-0.776, 0.696) | 0.777 | 0.987 |
| CXCL10 | -0.127 (-0.772, 0.680) | 0.726 | 0.987 |

Spearman’s rank correlation coefficients (*r*) with 95% confidence intervals (CIs) were used to assess associations between the 45 cytokines included in the analysis and IgG and IgM antibody levels. Raw *P* values and Benjamini-Hochberg (BH)-adjusted *P* values are shown. For multiple-testing correction, cytokine-antibody correlations were adjusted separately within each infection group, with 90 tests included in each BH correction. Antibody levels were represented by OD values measured at a 1:100 serum dilution. BH-adjusted *P* < 0.05 was considered statistically significant.

**Table S9.** Correlation analysis between serum cytokine levels and virus-specific IgG antibody levels in DENV-2-infected patients.

| Cytokine | Spearman’s r (95%CI) | Raw *P* | BH-adjusted *P* |
| --- | --- | --- | --- |
| TRAIL | -0.745 (-0.962, -0.216) | 0.013 | 0.284 |
| TNF-β | -0.806 (-0.975, -0.358) | 0.005 | 0.284 |
| IL-9 | -0.758 (-0.923, -0.208) | 0.011 | 0.284 |
| IL-1β | -0.752 (-0.975, -0.189) | 0.012 | 0.284 |
| CXCL1 | -0.733 (-0.914, -0.119) | 0.016 | 0.284 |
| CCL4 | -0.709 (-0.920, -0.119) | 0.022 | 0.325 |
| IL-12p40 | -0.673 (-0.903, -0.124) | 0.033 | 0.424 |
| IL-1Ra | -0.657 (-0.953, -0.019) | 0.039 | 0.441 |
| IL-18 | 0.612 (-0.132, 0.926) | 0.060 | 0.540 |
| CCL5 | -0.564 (-0.922, 0.031) | 0.090 | 0.734 |
| IL-2 | -0.512 (-0.843, 0.058) | 0.130 | 0.781 |
| GM-CSF | -0.529 (-0.950, 0.163) | 0.116 | 0.781 |
| SCF | -0.539 (-0.948, 0.183) | 0.108 | 0.781 |
| TNF-α | -0.492 (-0.834, 0.309) | 0.148 | 0.834 |
| HGF | -0.412 (-0.822, 0.231) | 0.236 | 0.880 |
| CCL27 | 0.333 (-0.547, 0.962) | 0.347 | 0.880 |
| LIF | -0.308 (-0.800, 0.403) | 0.387 | 0.880 |
| CXCL10 | -0.394 (-0.922, 0.409) | 0.260 | 0.880 |
| IL-15 | -0.304 (-0.900, 0.412) | 0.393 | 0.880 |
| IL-12p70 | -0.390 (-0.847, 0.258) | 0.265 | 0.880 |
| bFGF | -0.350 (-0.834, 0.406) | 0.322 | 0.880 |
| CXCL8 | 0.306 (-0.445, 0.829) | 0.389 | 0.880 |
| SCGF-β | 0.261 (-0.377, 0.761) | 0.467 | 0.880 |
| IL-6 | -0.273 (-0.887, 0.572) | 0.446 | 0.880 |
| IL-4 | -0.411 (-0.855, 0.309) | 0.238 | 0.880 |
| IL-3 | -0.275 (-0.843, 0.422) | 0.441 | 0.880 |
| sIL-2Rα | -0.418 (-0.975, 0.423) | 0.229 | 0.880 |
| CXCL12 | -0.248 (-0.776, 0.522) | 0.489 | 0.880 |
| IFN-γ | -0.407 (-0.886, 0.383) | 0.243 | 0.880 |
| CXCL9 | -0.316 (-0.961, 0.474) | 0.374 | 0.880 |
| CCL7 | -0.413 (-0.846, 0.259) | 0.235 | 0.880 |
| IL-13 | 0.230 (-0.605, 0.938) | 0.523 | 0.917 |
| PDGF-BB | -0.176 (-0.812, 0.547) | 0.627 | 0.925 |
| β-NGF | 0.201 (-0.459, 0.772) | 0.577 | 0.925 |
| IL-7 | 0.181 (-0.520, 0.794) | 0.617 | 0.925 |
| IL-1α | 0.188 (-0.519, 0.851) | 0.603 | 0.925 |
| G-CSF | -0.164 (-0.751, 0.597) | 0.650 | 0.944 |
| CCL2 | -0.042 (-0.650, 0.665) | 0.907 | 0.987 |
| CCL3 | 0.085 (-0.595, 0.737) | 0.815 | 0.987 |
| M-CSF | -0.079 (-0.772, 0.711) | 0.829 | 0.987 |
| IL-17A | -0.043 (-0.665, 0.746) | 0.906 | 0.987 |

**Table S9 (Continued).** Correlation analysis between serum cytokine levels and virus-specific IgG antibody levels in DENV-2-infected patients

| Cytokine | Spearman’s r (95%CI) | Raw *P* | BH-adjusted *P* |
| --- | --- | --- | --- |
| CCL11 | 0.030 (-0.689, 0.792) | 0.934 | 0.987 |
| IL-10 | 0.043 (-0.651, 0.717) | 0.906 | 0.987 |
| MIF | -0.127 (-0.750, 0.557) | 0.726 | 0.987 |
| IL-16 | 0.091 (-0.597, 0.868) | 0.803 | 0.987 |

Spearman’s rank correlation coefficients (*r*) with 95% confidence intervals (CIs) were used to assess associations between the 45 cytokines included in the analysis and IgG and IgM antibody levels. Raw *P* values and Benjamini-Hochberg (BH)-adjusted *P* values are shown. For multiple-testing correction, cytokine-antibody correlations were adjusted separately within each infection group, with 90 tests included in each BH correction. Antibody levels were represented by OD values measured at a 1:100 serum dilution. BH-adjusted *P* < 0.05 was considered statistically significant.

**Table S10.** Correlation analysis between serum cytokine levels and virus-specific IgM antibody levels in CHIKV-infected patients.

| Cytokine | Spearman’s r (95%CI) | Raw *P* | BH-adjusted *P* |
| --- | --- | --- | --- |
| IL-4 | 0.471 (0.026, 0.822) | 0.017 | 0.783 |
| IL-2 | 0.476 (0.064, 0.774) | 0.016 | 0.783 |
| IL-1β | 0.425 (-0.005, 0.763) | 0.030 | 0.908 |
| β-NGF | -0.140 (-0.536, 0.289) | 0.496 | 0.908 |
| TNF-β | 0.214 (-0.258, 0.633) | 0.294 | 0.908 |
| IL-13 | 0.335 (-0.062, 0.673) | 0.094 | 0.908 |
| IL-17A | 0.358 (-0.088, 0.737) | 0.072 | 0.908 |
| IL-18 | 0.169 (-0.291, 0.599) | 0.410 | 0.908 |
| CXCL10 | 0.212 (-0.201, 0.587) | 0.298 | 0.908 |
| IL-12p70 | 0.221 (-0.167, 0.595) | 0.279 | 0.908 |
| LIF | 0.228 (-0.264, 0.655) | 0.262 | 0.908 |
| CCL7 | 0.254 (-0.144, 0.611) | 0.210 | 0.908 |
| CXCL9 | 0.119 (-0.290, 0.502) | 0.561 | 0.908 |
| CCL3 | 0.090 (-0.359, 0.477) | 0.661 | 0.908 |
| CCL4 | 0.150 (-0.337, 0.569) | 0.464 | 0.908 |
| SCF | 0.277 (-0.163, 0.634) | 0.171 | 0.908 |
| CXCL12 | 0.106 (-0.316, 0.524) | 0.608 | 0.908 |
| TNF-α | 0.287 (-0.181, 0.660) | 0.155 | 0.908 |
| CCL2 | 0.348 (-0.026, 0.631) | 0.082 | 0.908 |
| IL-10 | 0.235 (-0.176, 0.604) | 0.248 | 0.908 |
| IL-12p40 | 0.220 (-0.247, 0.655) | 0.281 | 0.908 |
| CXCL8 | 0.275 (-0.138, 0.609) | 0.174 | 0.908 |
| CCL27 | 0.133 (-0.297, 0.555) | 0.518 | 0.908 |
| CCL11 | 0.147 (-0.265, 0.523) | 0.472 | 0.908 |
| bFGF | 0.235 (-0.225, 0.626) | 0.248 | 0.908 |
| G-CSF | 0.181 (-0.274, 0.591) | 0.376 | 0.908 |
| CXCL1 | 0.142 (-0.292, 0.533) | 0.490 | 0.908 |

**Table S10 (Continued).** Correlation analysis between serum cytokine levels and virus-specific IgM antibody levels in CHIKV-infected patients.

| Cytokine | Spearman’s r (95%CI) | Raw *P* | BH-adjusted *P* |
| --- | --- | --- | --- |
| IL-9 | 0.278 (-0.183, 0.661) | 0.169 | 0.908 |
| IFN-γ | 0.109 (-0.310, 0.517) | 0.595 | 0.908 |
| IL-1α | 0.220 (-0.202, 0.589) | 0.280 | 0.908 |
| HGF | 0.151 (-0.280, 0.528) | 0.461 | 0.908 |
| IL-1Ra | 0.307 (-0.129, 0.636) | 0.128 | 0.908 |
| IL-7 | 0.351 (-0.024, 0.625) | 0.079 | 0.908 |
| TRAIL | 0.302 (-0.111, 0.626) | 0.133 | 0.908 |
| IL-15 | -0.079 (-0.465, 0.314) | 0.703 | 0.917 |
| CCL5 | 0.072 (-0.356, 0.475) | 0.734 | 0.930 |
| IL-16 | -0.066 (-0.472, 0.347) | 0.749 | 0.936 |
| M-CSF | 0.029 (-0.440, 0.477) | 0.888 | 0.983 |
| sIL-2Rα | -0.032 (-0.487, 0.447) | 0.877 | 0.983 |
| PDGF-BB | 0.037 (-0.360, 0.411) | 0.857 | 0.983 |
| IL-3 | 0.037 (-0.386, 0.461) | 0.859 | 0.983 |
| MIF | 0.001 (-0.387, 0.378) | 0.996 | 0.999 |
| GM-CSF | 0.000 (-0.428, 0.410) | 0.999 | 0.999 |
| SCGF-β | -0.001 (-0.443, 0.415) | 0.996 | 0.999 |
| IL-6 | -0.005 (-0.426, 0.399) | 0.980 | 0.999 |

Spearman’s rank correlation coefficients (*r*) with 95% confidence intervals (CIs) were used to assess associations between the 45 cytokines included in the analysis and IgG and IgM antibody levels. Raw *P* values and Benjamini-Hochberg (BH)-adjusted *P* values are shown. For multiple-testing correction, cytokine-antibody correlations were adjusted separately within each infection group, with 90 tests included in each BH correction. Antibody levels were represented by OD values measured at a 1:100 serum dilution. BH-adjusted *P* < 0.05 was considered statistically significant.

**Table S11.** Correlation analysis between serum cytokine levels and virus-specific IgG antibody levels in CHIKV-infected patients.

| Cytokine | Spearman’s r (95%CI) | Raw *P* | BH-adjusted *P* |
| --- | --- | --- | --- |
| β-NGF | -0.233 (-0.581, 0.177) | 0.251 | 0.908 |
| IL-12p70 | 0.107 (-0.356, 0.508) | 0.604 | 0.908 |
| IL-13 | -0.134 (-0.496, 0.260) | 0.513 | 0.908 |
| IL-15 | -0.110 (-0.504, 0.327) | 0.593 | 0.908 |
| IL-16 | -0.099 (-0.521, 0.335) | 0.630 | 0.908 |
| IL-17A | -0.198 (-0.560, 0.211) | 0.333 | 0.908 |
| CXCL10 | 0.176 (-0.231, 0.545) | 0.390 | 0.908 |
| LIF | 0.101 (-0.345, 0.544) | 0.623 | 0.908 |
| IL-10 | 0.223 (-0.237, 0.622) | 0.274 | 0.908 |
| CCL2 | 0.118 (-0.257, 0.479) | 0.566 | 0.908 |
| MIF | 0.093 (-0.325, 0.499) | 0.650 | 0.908 |
| CCL3 | -0.123 (-0.475, 0.227) | 0.550 | 0.908 |
| PDGF-BB | 0.311 (-0.089, 0.644) | 0.122 | 0.908 |

**Table S11 (Continued).** Correlation analysis between serum cytokine levels and virus-specific IgG antibody levels in CHIKV-infected patients.

| Cytokine | Spearman’s r (95%CI) | Raw *P* | BH-adjusted *P* |
| --- | --- | --- | --- |
| CCL5 | 0.119 (-0.289, 0.491) | 0.570 | 0.908 |
| SCF | -0.089 (-0.486, 0.306) | 0.665 | 0.908 |
| SCGF-β | 0.271 (-0.109, 0.615) | 0.180 | 0.908 |
| CXCL12 | 0.184 (-0.277, 0.607) | 0.368 | 0.908 |
| CCL7 | -0.200 (-0.543, 0.182) | 0.327 | 0.908 |
| IL-9 | 0.090 (-0.345, 0.493) | 0.661 | 0.908 |
| IL-12p40 | 0.144 (-0.305, 0.557) | 0.484 | 0.908 |
| IFN-γ | -0.286 (-0.617, 0.110) | 0.156 | 0.908 |
| CCL11 | -0.186 (-0.551, 0.212) | 0.364 | 0.908 |
| bFGF | -0.112 (-0.496, 0.304) | 0.586 | 0.908 |
| G-CSF | -0.180 (-0.529, 0.187) | 0.380 | 0.908 |
| CXCL8 | -0.123 (-0.490, 0.245) | 0.549 | 0.908 |
| IL-1α | 0.088 (-0.338, 0.474) | 0.667 | 0.908 |
| IL-1β | -0.162 (-0.513, 0.225) | 0.429 | 0.908 |
| IL-1Ra | -0.086 (-0.458, 0.315) | 0.676 | 0.908 |
| TRAIL | 0.161 (-0.176, 0.460) | 0.431 | 0.908 |
| IL-2 | 0.268 (-0.169, 0.642) | 0.195 | 0.908 |
| sIL-2Rα | -0.131 (-0.514, 0.300) | 0.522 | 0.908 |
| IL-3 | 0.115 (-0.340, 0.507) | 0.585 | 0.908 |
| IL-4 | 0.128 (-0.311, 0.541) | 0.543 | 0.908 |
| CCL27 | 0.080 (-0.368, 0.534) | 0.698 | 0.917 |
| CXCL9 | -0.074 (-0.480, 0.336) | 0.718 | 0.924 |
| CCL4 | -0.059 (-0.452, 0.359) | 0.773 | 0.953 |
| M-CSF | 0.028 (-0.428, 0.493) | 0.893 | 0.983 |
| HGF | 0.019 (-0.371, 0.407) | 0.926 | 0.983 |
| GM-CSF | 0.026 (-0.395, 0.463) | 0.898 | 0.983 |
| TNF-β | -0.043 (-0.475, 0.408) | 0.834 | 0.983 |
| IL-6 | 0.019 (-0.377, 0.421) | 0.928 | 0.983 |
| TNF-α | 0.021 (-0.400, 0.443) | 0.918 | 0.983 |
| IL-18 | -0.037 (-0.434, 0.381) | 0.857 | 0.983 |
| IL-7 | 0.038 (-0.291, 0.398) | 0.855 | 0.983 |
| CXCL1 | 0.001 (-0.415, 0.434) | 0.997 | 0.999 |

Spearman’s rank correlation coefficients (*r*) with 95% confidence intervals (CIs) were used to assess associations between the 45 cytokines included in the analysis and IgG and IgM antibody levels. Raw *P* values and BH-adjusted *P* values are shown. For multiple-testing correction, cytokine-antibody correlations were adjusted separately within each infection group, with 90 tests included in each BH correction. Antibody levels were represented by OD values measured at a 1:100 serum dilution. BH-adjusted *P* < 0.05 was considered statistically significant.

**Table S12.** Correlation analysis between serum cytokine levels and Ct values in DENV-1-infected patients.

| Cytokine | Spearman’s r (95%CI) | Raw *P* | BH-adjusted *P* |
| --- | --- | --- | --- |
| IL-4 | -0.722 (-0.839, -0.520) | < 0.001 | 0.001 |
| TRAIL | -0.580 (-0.799, -0.258) | 0.001 | 0.025 |
| IL-1β | -0.567 (-0.814, -0.203) | 0.002 | 0.025 |
| CCL2 | -0.423 (-0.703, -0.065) | 0.022 | 0.169 |
| IL-10 | 0.454 (0.095, 0.737) | 0.015 | 0.169 |
| HGF | -0.415 (-0.696, -0.077) | 0.028 | 0.169 |
| SCF | -0.410 (-0.714, -0.034) | 0.030 | 0.169 |
| G-CSF | -0.436 (-0.715, -0.049) | 0.023 | 0.169 |
| sIL-2Rα | 0.370 (0.020, 0.658) | 0.053 | 0.234 |
| bFGF | -0.375 (-0.705, 0.033) | 0.049 | 0.234 |
| IL-7 | -0.357 (-0.660, 0.016) | 0.057 | 0.234 |
| IL-1α | -0.347 (-0.671, 0.062) | 0.066 | 0.244 |
| CXCL12 | -0.347 (-0.678, 0.044) | 0.070 | 0.244 |
| CXCL9 | 0.342 (-0.081, 0.643) | 0.081 | 0.244 |
| IL-12p70 | 0.335 (-0.079, 0.688) | 0.081 | 0.244 |
| IL-1Ra | -0.296 (-0.626, 0.112) | 0.134 | 0.356 |
| CCL11 | -0.296 (-0.669, 0.128) | 0.126 | 0.356 |
| MIF | -0.266 (-0.586, 0.141) | 0.171 | 0.385 |
| IL-17A | -0.267 (-0.628, 0.124) | 0.169 | 0.385 |
| IL-16 | -0.271 (-0.646, 0.157) | 0.164 | 0.385 |
| IL-13 | -0.242 (-0.608, 0.175) | 0.215 | 0.449 |
| PDGF-BB | -0.229 (-0.562, 0.171) | 0.232 | 0.449 |
| CCL7 | -0.230 (-0.594, 0.163) | 0.239 | 0.449 |
| IL-2 | -0.237 (-0.612, 0.191) | 0.225 | 0.449 |
| CXCL10 | -0.213 (-0.527, 0.154) | 0.268 | 0.465 |
| CCL27 | 0.212 (-0.226, 0.568) | 0.269 | 0.465 |
| IL-3 | 0.205 (-0.217, 0.590) | 0.287 | 0.479 |
| CXCL1 | -0.190 (-0.551, 0.220) | 0.334 | 0.536 |
| IL-15 | 0.172 (-0.181, 0.463) | 0.371 | 0.576 |
| IFN-γ | 0.135 (-0.229, 0.495) | 0.485 | 0.727 |
| CCL4 | -0.123 (-0.495, 0.279) | 0.533 | 0.749 |
| GM-CSF | 0.130 (-0.229, 0.469) | 0.519 | 0.749 |
| SCGF-β | 0.104 (-0.330, 0.523) | 0.598 | 0.815 |
| LIF | -0.074 (-0.460, 0.353) | 0.708 | 0.826 |
| IL-18 | 0.090 (-0.296, 0.450) | 0.647 | 0.826 |
| TNF-β | -0.075 (-0.449, 0.298) | 0.705 | 0.826 |
| CCL3 | -0.078 (-0.432, 0.317) | 0.693 | 0.826 |
| CCL5 | 0.066 (-0.311, 0.434) | 0.734 | 0.826 |
| M-CSF | -0.094 (-0.407, 0.236) | 0.634 | 0.826 |
| IL-9 | -0.072 (-0.437, 0.305) | 0.716 | 0.826 |
| β-NGF | 0.038 (-0.400, 0.443) | 0.847 | 0.911 |

**Table S12 (Continued).** Correlation analysis between serum cytokine levels and Ct values in DENV-1-infected patients.

| Cytokine | Spearman’s r (95%CI) | Raw *P* | BH-adjusted *P* |
| --- | --- | --- | --- |
| CXCL8 | 0.037 (-0.326, 0.414) | 0.850 | 0.911 |
| IL-6 | 0.027 (-0.321, 0.367) | 0.891 | 0.933 |
| TNF-α | -0.019 (-0.413, 0.362) | 0.925 | 0.946 |
| IL-12p40 | -0.006 (-0.391, 0.373) | 0.975 | 0.975 |

Spearman’s rank correlation coefficients (*r*) with 95% confidence intervals (CIs), raw P values, and BH-adjusted *P* values are shown. Correlations were assessed for 45 cytokines included in the analysis. For multiple-testing correction, 45 tests were included in the BH correction. A BH-adjusted *P* value < 0.05 was considered statistically significant.

**Table S13.** Comparison of serum cytokine levels between DENV-1 patients stratified by the median Ct value.

| Cytokine | U statistic | HL (95% CI) | Raw *P* | BH-adjusted *P* |
| --- | --- | --- | --- | --- |
| IL-4 | 160.000 | 3.270 (1.760, 4.860) | 0.001 | 0.045 |
| IL-7 | 159.000 | 38.873 (0.000, 58.850) | 0.017 | 0.202 |
| IL-10 | 46.000 | -11.780 (-26.967, -2.340) | 0.018 | 0.202 |
| G-CSF | 140.000 | 27.600 (3.025, 37.360) | 0.018 | 0.202 |
| TRAIL | 140.000 | 136.685 (-1.128, 300.228) | 0.057 | 0.382 |
| IL-1α | 149.000 | 28.660 (0.000, 46.261) | 0.052 | 0.382 |
| HGF | 136.500 | 160.740 (-27.640, 390.425) | 0.080 | 0.382 |
| IL-1β | 137.500 | 7.205 (-2.040, 15.900) | 0.072 | 0.382 |
| bFGF | 137.000 | 66.350 (-0.106, 119.420) | 0.075 | 0.382 |
| CCL11 | 136.000 | 73.200 (-9.780, 144.405) | 0.085 | 0.382 |
| sIL-2Rα | 62.500 | -10.105 (-26.210, 3.867) | 0.108 | 0.439 |
| IL-13 | 132.500 | 4.500 (-2.290, 9.740) | 0.117 | 0.439 |
| IL-17A | 128.500 | 18.715 (-8.320, 41.783) | 0.167 | 0.540 |
| CCL3 | 128.500 | 1.470 (-0.610, 5.720) | 0.168 | 0.540 |
| IL-2 | 127.500 | 2.260 (-1.190, 4.530) | 0.181 | 0.543 |
| IL-16 | 126.000 | 28.705 (-22.480, 92.682) | 0.206 | 0.545 |
| SCF | 126.500 | 76.490 (-24.510, 169.310) | 0.198 | 0.545 |
| CXCL12 | 124.000 | 771.905 (-517.014, 1945.390) | 0.241 | 0.602 |
| CCL2 | 126.500 | 70.480 (-53.191, 192.962) | 0.359 | 0.619 |
| MIF | 118.000 | 568.010 (-827.830, 1074.180) | 0.370 | 0.619 |
| IL-15 | 90.000 | 0.000 (-360.450, 0.000) | 0.399 | 0.619 |
| CCL7 | 120.000 | 2.420 (-2.980, 5.320) | 0.322 | 0.619 |
| CXCL8 | 125.000 | 13.733 (-10.807, 32.393) | 0.377 | 0.619 |
| IL-3 | 81.500 | -0.376 (-1.750, 0.520) | 0.314 | 0.619 |
| IFN-γ | 84.000 | 0.000 (-23.290, 0.000) | 0.342 | 0.619 |
| CXCL1 | 117.000 | 177.695 (-204.860, 300.169) | 0.395 | 0.619 |
| GM-CSF | 69.500 | -1.970 (-10.000, 2.815) | 0.307 | 0.619 |
| IL-12p70 | 73.500 | -1.090 (-10.010, 3.320) | 0.267 | 0.619 |
| PDGF-BB | 126.000 | 1531.650 (-2421.510, 4054.170) | 0.371 | 0.619 |

| Cytokine | U statistic | HL (95% CI) | Raw *P* | BH-adjusted *P* |
| --- | --- | --- | --- | --- |
| CXCL10 | 119.000 | 1173.075 (-5193.840, 5096.855) | 0.556 | 0.807 |
| TNF-α | 111.500 | 16.600 (-17.060, 44.591) | 0.548 | 0.807 |
| CCL4 | 109.000 | 42.130 (-52.680, 75.890) | 0.629 | 0.828 |
| IL-18 | 86.500 | 0.000 (-36.150, 24.080) | 0.613 | 0.828 |
| M-CSF | 87.000 | -0.540 (-11.900, 6.870) | 0.629 | 0.828 |
| CXCL9 | 81.000 | -77.130 (-2280.790, 229.620) | 0.644 | 0.828 |
| IL-1Ra | 98.500 | 0.000 (-1428.394, 1336.350) | 0.733 | 0.916 |
| SCGF-β | 91.000 | -3196.930 (-82055.690, 62964.820) | 0.765 | 0.930 |
| CCL5 | 100.000 | 602.805 (-4708.130, 3688.170) | 0.844 | 0.931 |
| β-NGF | 109.000 | 0.775 (-2.339, 3.090) | 0.878 | 0.931 |
| TNF-β | 101.500 | 15.875 (-60.730, 66.370) | 0.890 | 0.931 |
| IL-9 | 103.000 | 56.845 (-147.882, 141.730) | 0.836 | 0.931 |
| IL-6 | 92.000 | 0.000 (-5.160, 3.040) | 0.800 | 0.931 |
| LIF | 102.500 | 14.055 (-61.030, 110.790) | 0.853 | 0.931 |
| CCL27 | 104.000 | -22.380 (-721.481, 655.275) | 0.983 | 0.983 |
| IL-12p40 | 96.500 | 0.000 (-87.570, 45.230) | 0.963 | 0.983 |

**Table S13 (Continued).** Comparison of serum cytokine levels between DENV-1 patients stratified by the median Ct value.

Patients were divided into low-Ct and high-Ct groups according to the median Ct value. Cytokine levels were compared using the Mann-Whitney U test. U statistics, Hodges-Lehmann (HL) estimates (low Ct - high Ct) with 95% confidence intervals (CIs), raw *P* values, and BH-adjusted *P* values are shown. The analysis included 45 cytokines, corresponding to 45 tests in the BH correction. BH-adjusted *P* < 0.05 was considered statistically significant.

**Table S14.** Comparison of serum cytokine levels between male and female patients infected with DENV-2.

| Cytokine | U statistic | HL (95% CI) | Raw *P* | BH-adjusted *P* |
| --- | --- | --- | --- | --- |
| PDGF-BB | 1.000 | -2545.580 (-4063.370, -949.930) | 0.016 | 0.240 |
| MIF | 1.000 | -1075.260 (-3236.240, -302.740) | 0.016 | 0.240 |
| CXCL12 | 1.000 | -1938.060 (-2282.630, -526.940) | 0.016 | 0.240 |
| CCL2 | 6.000 | -105.740 (-197.430, 23.440) | 0.222 | 0.624 |
| IL-16 | 4.000 | -61.990 (-130.000, 12.050) | 0.095 | 0.624 |
| IL-15 | 5.000 | -485.620 (-1073.673, 0.000) | 0.119 | 0.624 |
| IL-10 | 20.000 | 8.290 (0.000, 68.050) | 0.136 | 0.624 |
| CXCL8 | 5.500 | -13.450 (-30.000, 6.130) | 0.161 | 0.624 |
| IL-7 | 5.500 | -17.433 (-84.540, 0.000) | 0.147 | 0.624 |
| CCL4 | 6.000 | -96.930 (-289.020, 21.790) | 0.222 | 0.624 |
| IL-2 | 5.500 | -2.890 (-7.920, 2.830) | 0.172 | 0.624 |
| SCF | 4.000 | -113.070 (-304.880, 18.240) | 0.095 | 0.624 |

**Table S14 (Continued).** Comparison of serum cytokine levels between male and female patients infected with DENV-2.

| Cytokine | U statistic | HL (95% CI) | Raw *P* | BH-adjusted *P* |
| --- | --- | --- | --- | --- |
| G-CSF | 5.500 | -21.370 (-52.870, 9.990) | 0.173 | 0.624 |
| CCL27 | 6.000 | -291.520 (-1319.260, 214.000) | 0.222 | 0.624 |
| CCL11 | 5.000 | -96.190 (-376.370, 14.350) | 0.151 | 0.624 |
| bFGF | 6.000 | -140.120 (-230.260, 25.350) | 0.205 | 0.624 |
| IFN-γ | 7.000 | -20.990 (-60.330, 0.690) | 0.265 | 0.701 |
| IL-17A | 7.000 | -16.940 (-91.050, 8.410) | 0.290 | 0.704 |
| IL-12p70 | 7.500 | -9.680 (-15.830, 14.350) | 0.344 | 0.704 |
| TNF-α | 7.500 | -43.040 (-71.545, 8.890) | 0.340 | 0.704 |
| IL-6 | 7.000 | -5.950 (-10.880, 2.490) | 0.310 | 0.704 |
| LIF | 7.500 | -66.950 (-238.770, 56.930) | 0.340 | 0.704 |
| HGF | 8.000 | -167.650 (-222.160, 54.510) | 0.396 | 0.775 |
| CXCL1 | 8.000 | -474.640 (-786.120, 196.860) | 0.421 | 0.789 |
| IL-1β | 8.500 | -11.780 (-26.450, 6.240) | 0.461 | 0.830 |
| GM-CSF | 9.000 | -6.810 (-23.520, 9.640) | 0.530 | 0.850 |
| IL-13 | 9.000 | -2.290 (-6.640, 6.430) | 0.521 | 0.850 |
| SCGF-β | 9.000 | -23618.600 (-183121.030, 62048.090) | 0.548 | 0.850 |
| IL-9 | 9.000 | -195.270 (-572.790, 134.300) | 0.548 | 0.850 |
| β-NGF | 9.500 | -1.542 (-13.840, 1.940) | 0.599 | 0.862 |
| CXCL9 | 10.000 | -71.050 (-259.840, 95.850) | 0.675 | 0.862 |
| CCL5 | 10.000 | -5254.680 (-7636.190, 2980.030) | 0.690 | 0.862 |
| TRAIL | 15.000 | -28.480 (-198.180, 222.760) | 0.690 | 0.862 |
| IL-18 | 10.000 | 5.150 (-46.400, 35.320) | 0.690 | 0.862 |
| TNF-β | 10.000 | -69.960 (-196.560, 35.210) | 0.690 | 0.862 |
| IL-1Ra | 9.500 | -1754.080 (-3989.800, 1963.300) | 0.600 | 0.862 |
| CCL3 | 10.500 | -0.120 (-2.600, 1.890) | 0.753 | 0.892 |
| IL-4 | 14.500 | 0.000 (-6.480, 4.080) | 0.747 | 0.892 |
| sIL-2Rα | 11.000 | -1.260 (-19.370, 10.750) | 0.841 | 0.946 |
| CCL7 | 11.000 | -2.850 (-8.030, 4.990) | 0.834 | 0.946 |
| IL-3 | 11.500 | -0.402 (-3.630, 1.350) | 0.916 | 0.981 |
| IL-12p40 | 11.500 | -94.290 (-196.130, 139.520) | 0.916 | 0.981 |
| M-CSF | 12.000 | -0.540 (-11.140, 34.770) | 1.000 | 1.000 |
| CXCL10 | 12.000 | -5708.780 (-12841.220, 8151.180) | 1.000 | 1.000 |
| IL-1α | 12.000 | 0.000 (-69.970, 3.701) | 1.000 | 1.000 |

Cytokine levels were compared between male and female patients using the Mann-Whitney U test. U statistics, Hodges-Lehmann (HL) estimates (male - female) with 95% confidence intervals (CIs), raw *P* values, and BH-adjusted *P* values are shown. The analysis included 45 cytokines, corresponding to 45 tests in the BH correction. BH-adjusted *P* < 0.05 was considered statistically significant.

**Table S15.** Comparison of serum cytokine levels between male and female patients infected with CHIKV.

| Cytokine | U statistic | HL (95% CI) | Raw *P* | BH-adjusted *P* |
| --- | --- | --- | --- | --- |
| IL-9 | 42.000 | -185.420 (-322.540, -4.520) | 0.038 | 0.450 |
| TNF-β | 40.500 | -94.780 (-153.220, -5.950) | 0.031 | 0.450 |
| sIL-2Rα | 42.500 | -21.440 (-38.460, -1.870) | 0.040 | 0.450 |
| SCF | 42.500 | -78.390 (-155.320, -4.446) | 0.040 | 0.450 |
| IL-16 | 114.000 | 99.870 (-29.190, 201.470) | 0.107 | 0.602 |
| M-CSF | 50.500 | -11.660 (-19.290, 0.550) | 0.102 | 0.602 |
| IL-1α | 115.000 | 14.540 (0.000, 69.970) | 0.094 | 0.602 |
| CCL5 | 45.000 | -8428.740 (-12299.919, 52.356) | 0.085 | 0.602 |
| IL-7 | 111.000 | 17.433 (0.000, 38.873) | 0.136 | 0.612 |
| CXCL1 | 52.500 | -373.730 (-612.870, 83.230) | 0.126 | 0.612 |
| CCL4 | 62.500 | -65.690 (-181.890, 41.070) | 0.311 | 0.651 |
| LIF | 110.000 | 74.210 (-31.192, 156.880) | 0.160 | 0.651 |
| IL-10 | 106.500 | 2.290 (-4.630, 6.970) | 0.218 | 0.651 |
| IL-18 | 65.000 | -23.280 (-91.850, 31.290) | 0.378 | 0.651 |
| IL-17A | 100.500 | 8.490 (-16.860, 37.830) | 0.362 | 0.651 |
| SCGF-β | 64.000 | -53468.770 (-132411.190, 44735.120) | 0.350 | 0.651 |
| IL-15 | 58.000 | -301.590 (-926.633, 44.210) | 0.195 | 0.651 |
| TNF-α | 57.000 | -24.490 (-52.170, 8.350) | 0.192 | 0.651 |
| IL-12p70 | 99.000 | 3.520 (-8.710, 11.550) | 0.405 | 0.651 |
| IL-12p40 | 65.500 | -42.580 (-135.870, 45.230) | 0.387 | 0.651 |
| TRAIL | 99.500 | 8.760 (-51.790, 114.000) | 0.392 | 0.651 |
| IL-4 | 93.000 | 1.510 (-1.510, 3.840) | 0.326 | 0.651 |
| CCL27 | 101.000 | 117.460 (-493.065, 831.820) | 0.350 | 0.651 |
| IL-3 | 94.000 | 1.000 (-1.010, 2.161) | 0.304 | 0.651 |
| CCL11 | 57.500 | -70.390 (-127.600, 26.040) | 0.203 | 0.651 |
| IFN-γ | 60.500 | -16.380 (-37.076, 11.504) | 0.257 | 0.651 |
| HGF | 99.000 | 77.590 (-154.310, 370.150) | 0.405 | 0.651 |
| GM-CSF | 59.000 | -5.880 (-14.937, 4.480) | 0.232 | 0.651 |
| IL-6 | 68.000 | -6.340 (-15.570, 2.580) | 0.466 | 0.723 |
| G-CSF | 95.000 | 16.380 (-17.280, 54.450) | 0.532 | 0.750 |
| CXCL8 | 95.000 | 23.283 (-25.793, 62.323) | 0.525 | 0.750 |
| MIF | 95.000 | 433.190 (-1074.020, 1538.592) | 0.533 | 0.750 |
| bFGF | 72.500 | -22.030 (-66.390, 48.860) | 0.621 | 0.823 |
| PDGF-BB | 73.000 | -836.590 (-4491.549, 2959.047) | 0.640 | 0.823 |
| CCL7 | 91.000 | 1.150 (-6.500, 10.890) | 0.677 | 0.823 |
| IL-1β | 73.000 | -1.970 (-10.110, 6.470) | 0.640 | 0.823 |
| IL-13 | 74.000 | -2.030 (-6.640, 4.900) | 0.675 | 0.823 |
| CXCL9 | 74.500 | -170.600 (-724.527, 293.070) | 0.697 | 0.825 |
| CCL3 | 86.500 | 3.670 (-2.200, 9.340) | 0.856 | 0.896 |

**Table S15 (Continued).** Comparison of serum cytokine levels between male and female patients infected with CHIKV.

| Cytokine | U statistic | HL (95% CI) | Raw *P* | BH-adjusted *P* |
| --- | --- | --- | --- | --- |
| CCL2 | 87.000 | -2.200 (-144.330, 134.240) | 0.835 | 0.896 |
| IL-1Ra | 87.000 | 0.000 (-907.850, 1516.450) | 0.835 | 0.896 |
| IL-2 | 71.000 | -1.110 (-3.950, 3.450) | 0.845 | 0.896 |
| CXCL12 | 88.000 | -67.670 (-1247.066, 1359.680) | 0.795 | 0.896 |
| CXCL10 | 80.000 | -2281.340 (-7212.720, 4707.780) | 0.917 | 0.917 |
| β-NGF | 80.000 | -0.770 (-4.230, 5.390) | 0.917 | 0.917 |

Cytokine levels were compared between male and female patients using the Mann-Whitney U test. U statistics, Hodges-Lehmann (HL) estimates (male - female) with 95% confidence intervals (CIs), raw *P* values, and BH-adjusted *P* values are shown. The analysis included 45 cytokines, corresponding to 45 tests in the BH correction. BH-adjusted *P* < 0.05 was considered statistically significant.
